# Supplementary figures and images for: Predicting stimulation-dependent enhancer-promoter interactions from ChIP-Seq time course data
Source: PeerJ. 2017 Sep 28;5:e3742. doi: 10.7717/peerj.3742 (PMC5623311; doi:10.7717/peerj.3742)

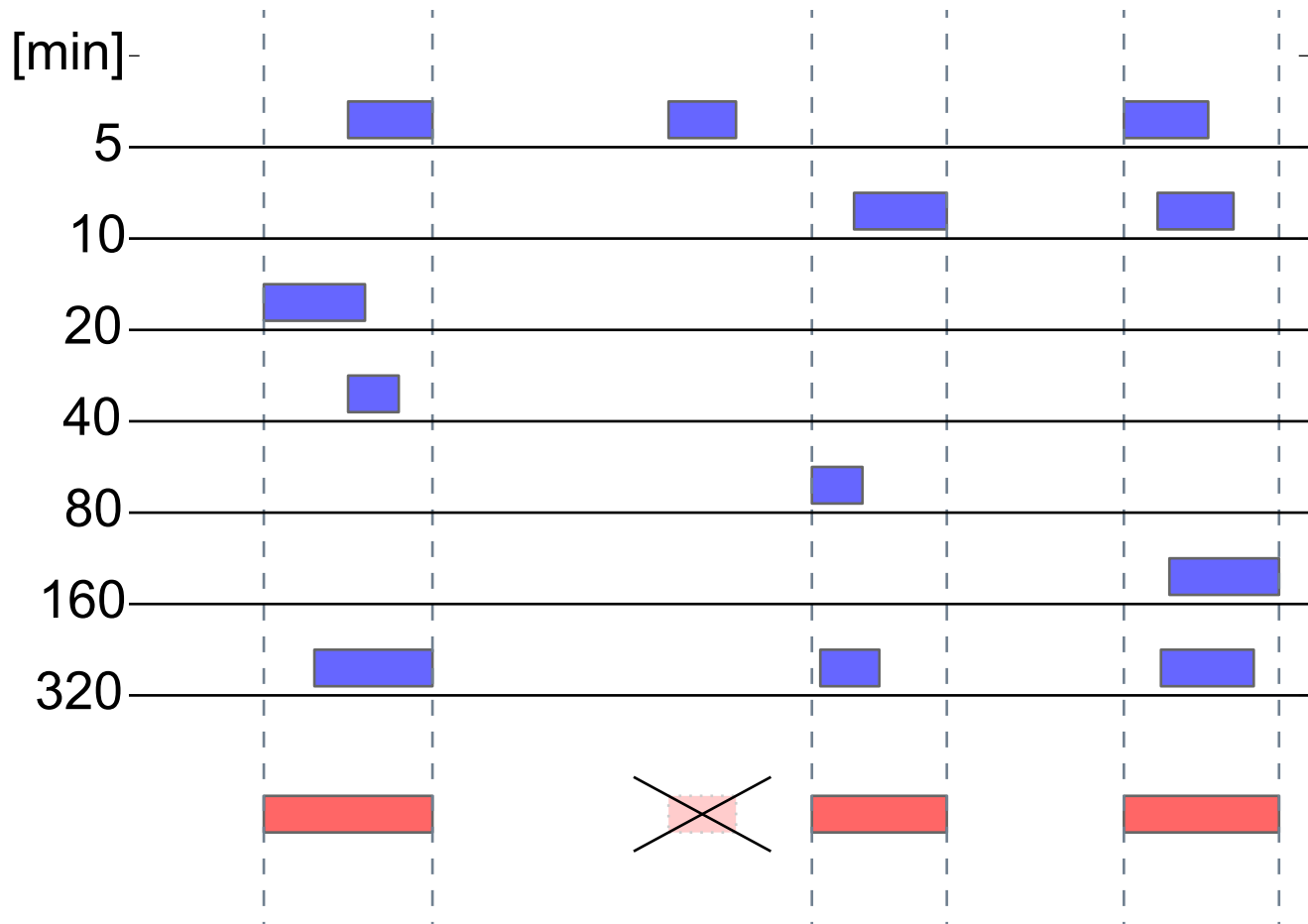

Supplement: Figure S1 — Cartoon shows the process of merging individual MACS-called peaks with the objective of finding approximate locations of time persistent ER-α bindings. In the process MACS-detected time varying peaks from [0], 5, . . . , 320 min time points (0 is optional and by default not included) which co-occur at least twice across time points are merged by union operation to produce the approximate consensus locations of a single binding. The single occurrences of peaks are discarded. [file peerj-05-3742-s001.pdf]

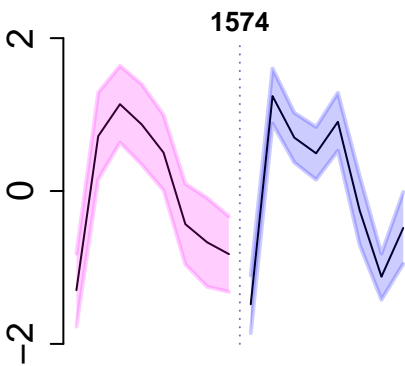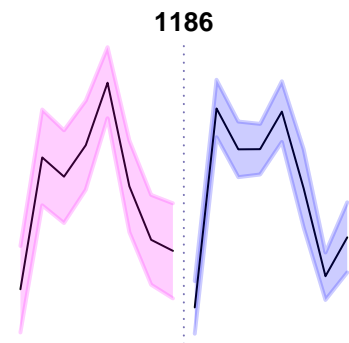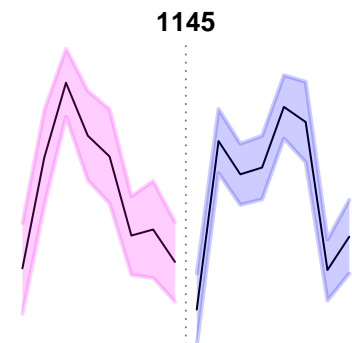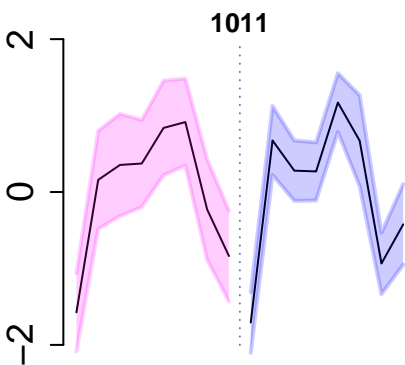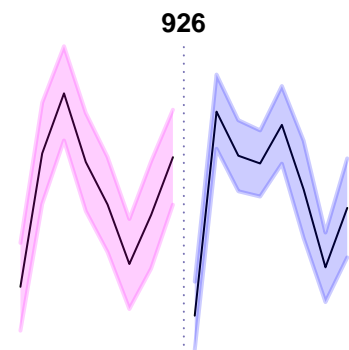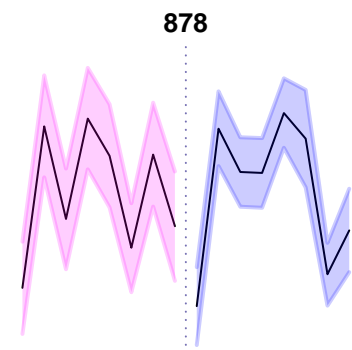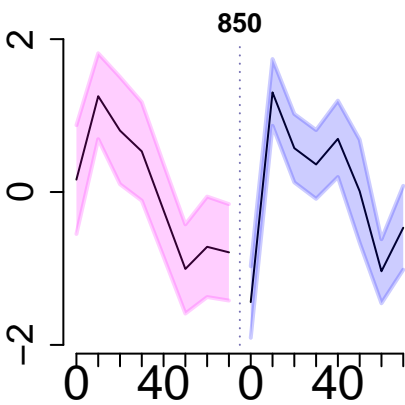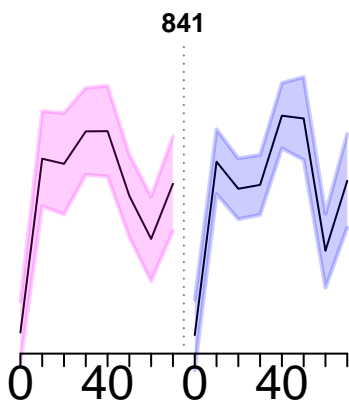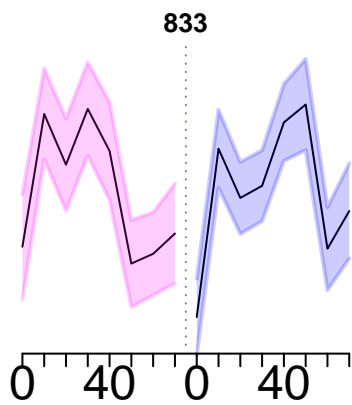

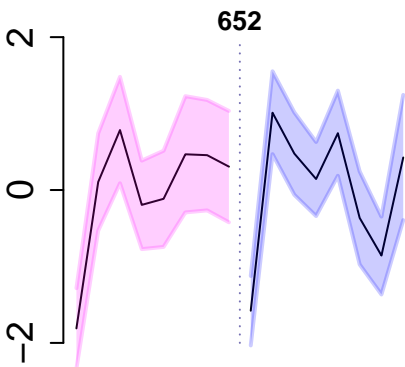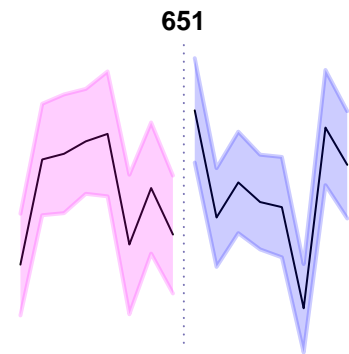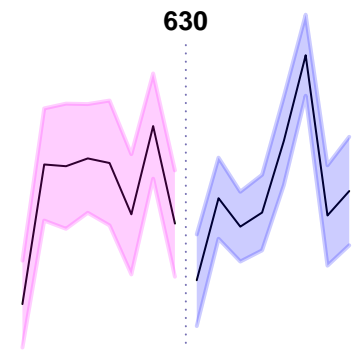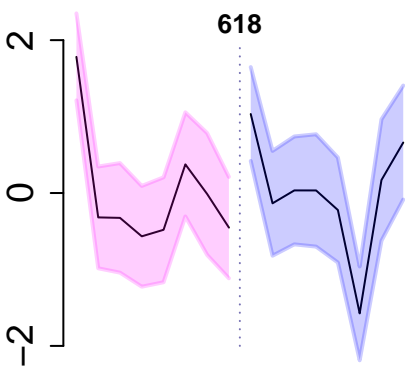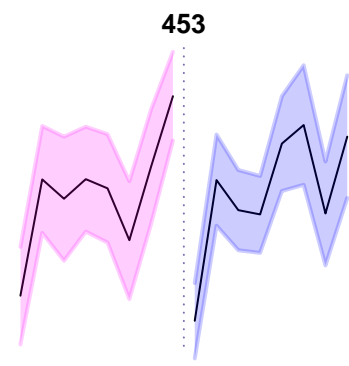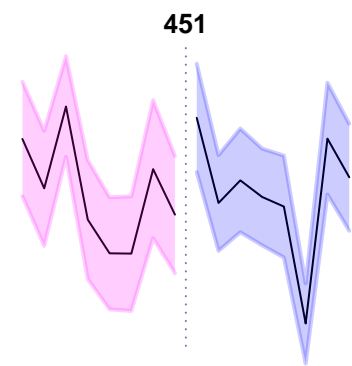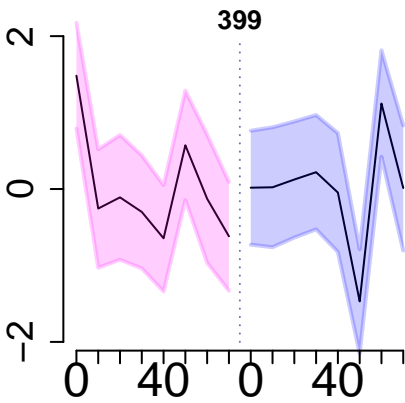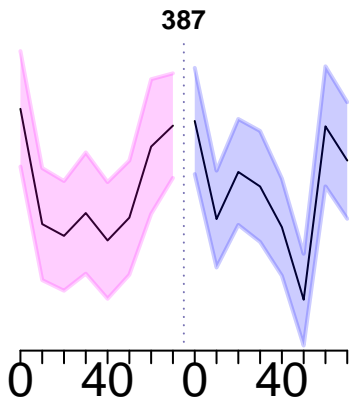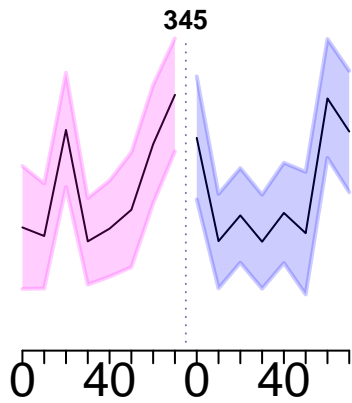

Supplement: Figure S2 — Figure shows the clustering of the joint time course of Pol II and ER-α at enhancers with Affinity Propagation. The clustering involves only the time series which individually possess a sum of at least 200 tags across all time point. [file peerj-05-3742-s002.pdf]

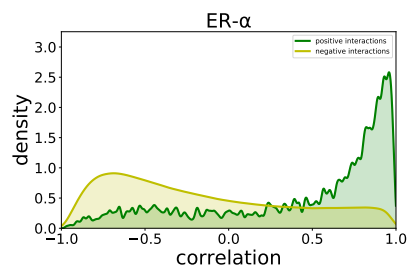

(a)

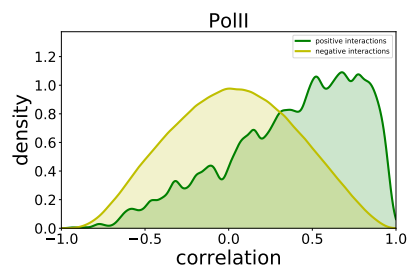

(b)

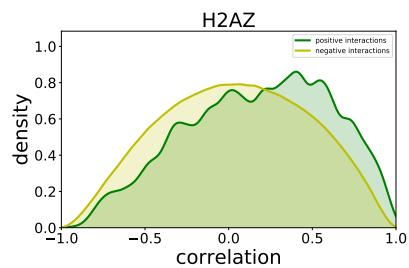

(c)

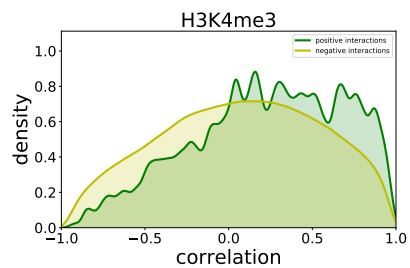

(d)

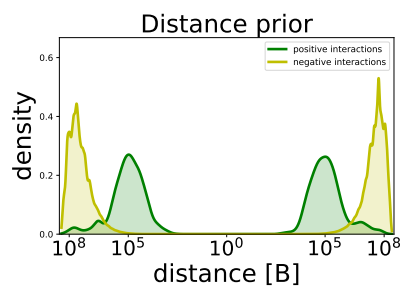

(e)

Supplement: Figure S3 — The graphs (A–D) show positive (green) and negative (yellow) distributions of correlations between time series of 300 bp-upstream-extended- gene regions and enhancer bodies for ER- α, PolII, H2AZ and H3K4me3 collected across all 23 chromosomes. The figure (E) shows the distribution of genomic distances between centres of distal enhancers and 300 bp-upstream-shifted-TSS of genes. The set of positive and negative pairs was constructed using 300 bp- upstream-extended-genes and distal enhancers. [file peerj-05-3742-s003.pdf]

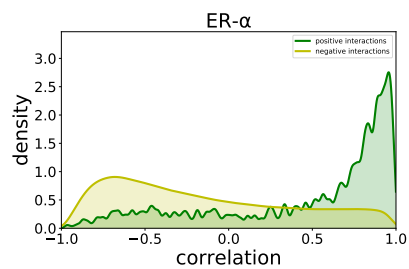

(a)

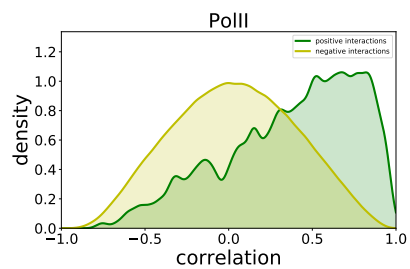

(b)

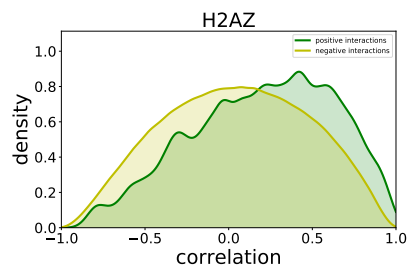

(c)

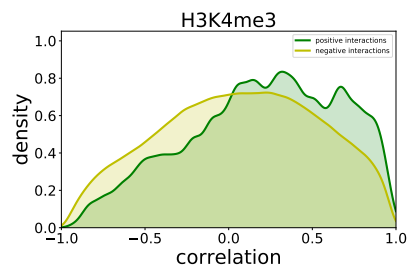

(d)

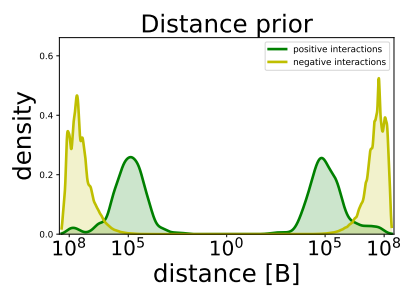

(e)

Supplement: Figure S4 — The graphs (A–D) show positive (green) and negative (yellow) distributions of correlations between time series of 300 bp-upstream-extended- gene regions and enhancer bodies for ER- α, PolII, H2AZ and H3K4me3 collected across all odd chromosomes. The figure (E) shows the distribution of genomic distances between centres of distal enhancers and 1,500 bp-upstream-shifted-TSS of genes. The set of positive and negative pairs was constructed using 1,500 bp- upstream-extended-genes and distal enhancers. [file peerj-05-3742-s004.pdf]

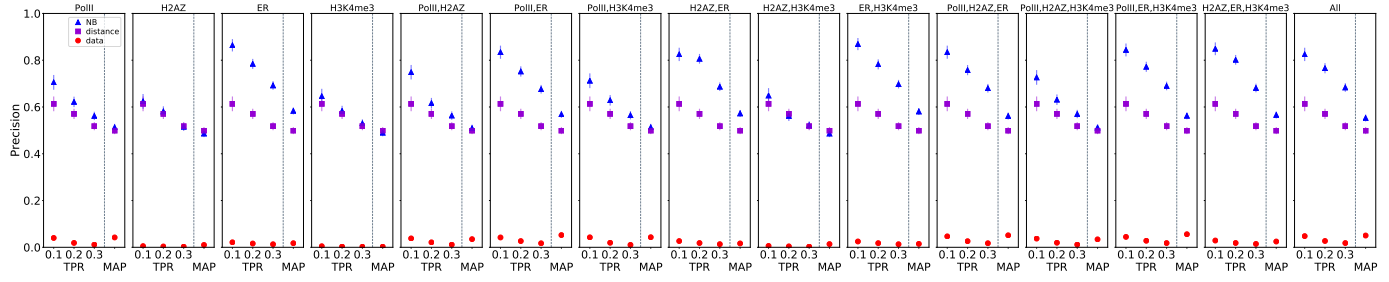

(a) training data performance

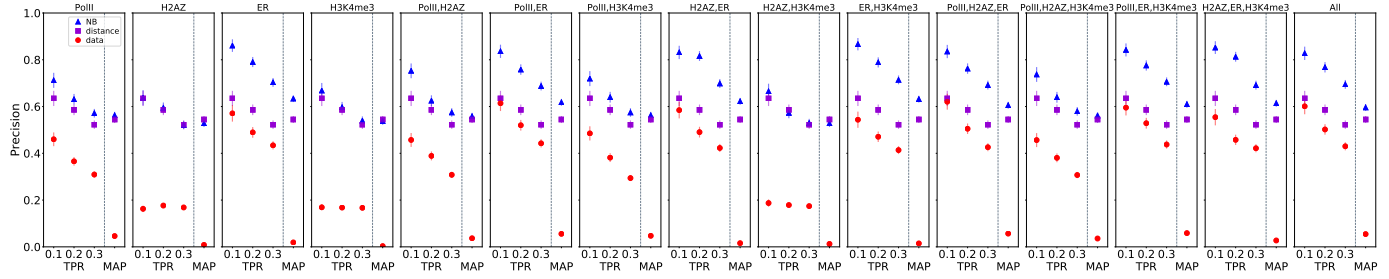

(b) intra-domain performance

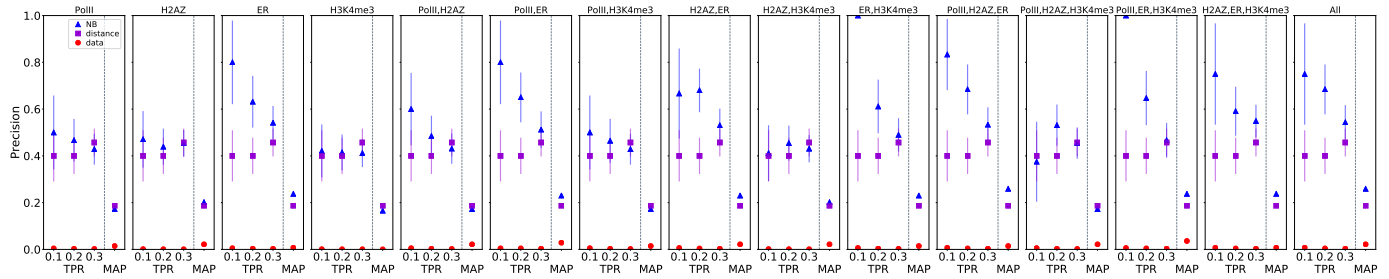

(c) inter-domain performance

Supplement: Figure S5 — Figure shows the comparison of performance of the NB model on odd chromosomes (training data) measured by Precision-TPR and MAP scores. The Precision-TPR curves show the accuracy of the predictions with the highest 10%, 20%, 30% scores i.e., posterior probabilities. The second and the third rows stratify predictions at each of the thresholds into those which take place within domains and those involving inter-domain contacts. The set of positive and negative pairs for the first model was constructed using 300 bp-upstream- extended-genes and distal enhancers. The correlation-based attributes of the two models were estimated using signals (time series) aggregated over 300bp- upstream-extended-genes, and distal enhancer bodies. The separation-based feature was estimated from 300bp-upstream-shifted TSS to the centres of the ER-α enhancers. [file peerj-05-3742-s005.pdf]

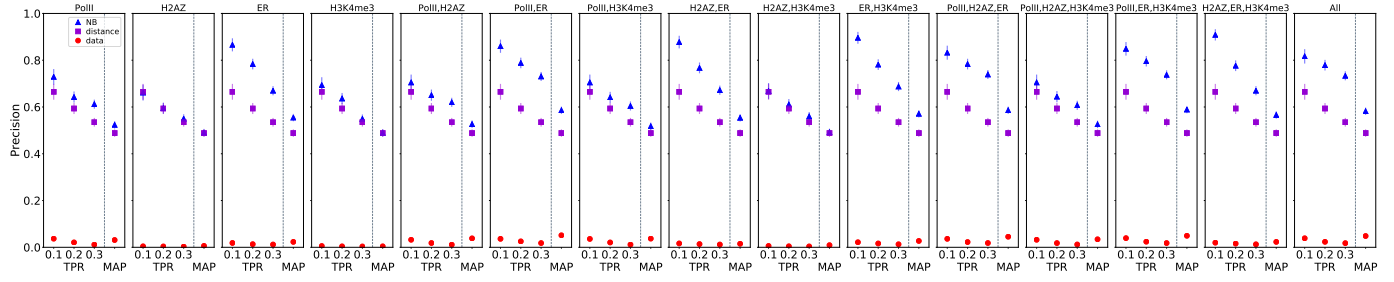

(a) training data performance

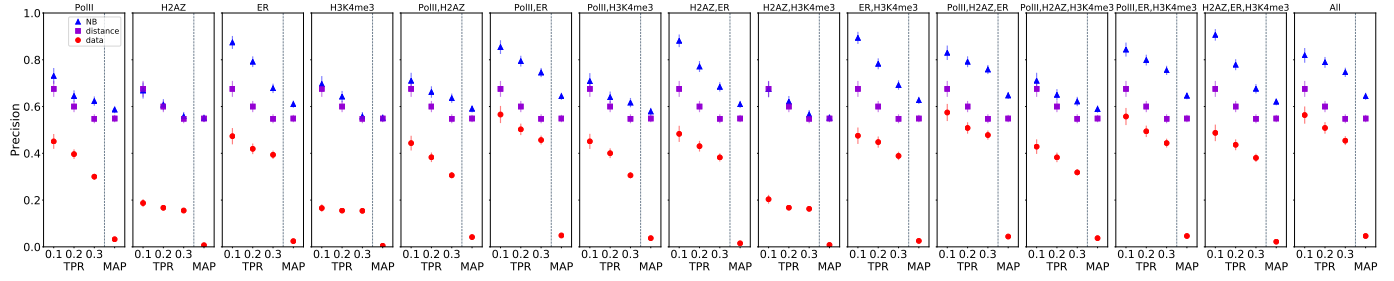

(b) intra-domain performance

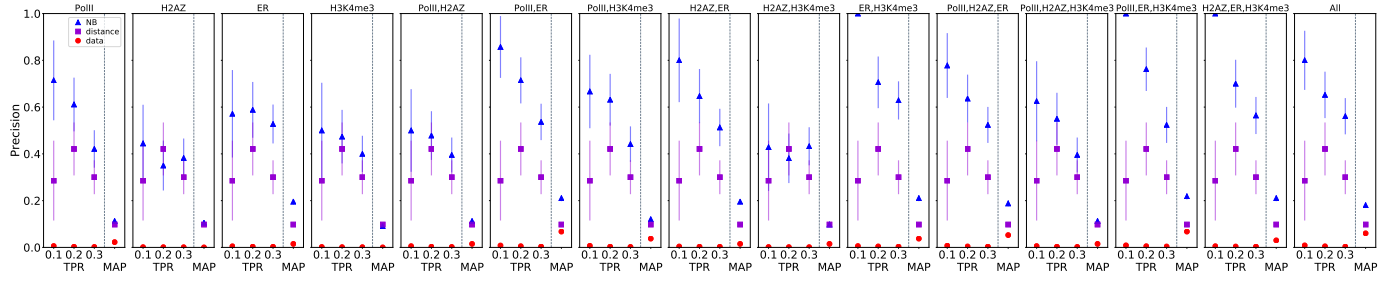

(c) inter-domain performance

Supplement: Figure S6 — Figure shows the comparison of performance of the NB model on even chromosomes (test data) measured by Precision-TPR and MAP scores. The Precision-TPR curves show the accuracy of the predictions with the highest 10%, 20%, 30% scores i.e. posterior probabilities. The second and the third rows stratify predictions at each of the thresholds into those which take place within domains and those involving inter-domain contacts. The set of positive and negative pairs for the first model was constructed using 300bp-upstream- extended-genes and distal enhancers. The correlation-based attributes of the two models were estimated using signals (time series) aggregated over 300bp- upstream-extended-genes, and distal enhancer bodies. The separation-based feature was estimated from 300 bp-upstream-shifted TSS to the centres of the ER-α enhancers. [file peerj-05-3742-s006.pdf]

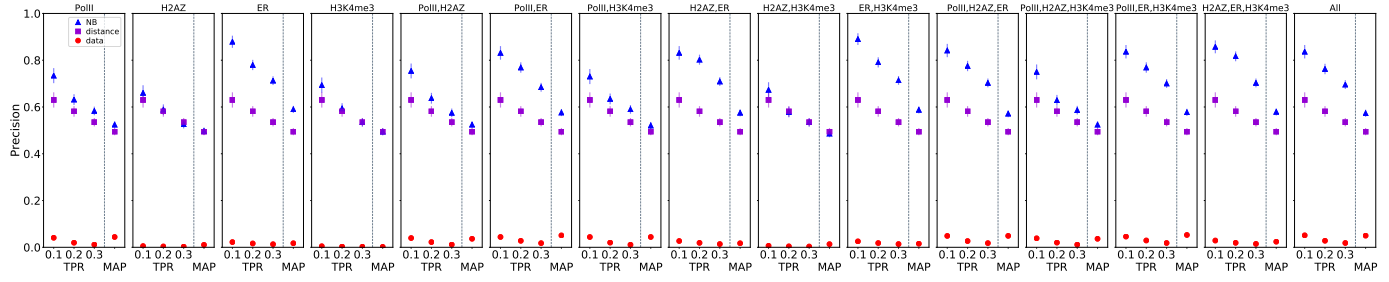

(a) training data performance

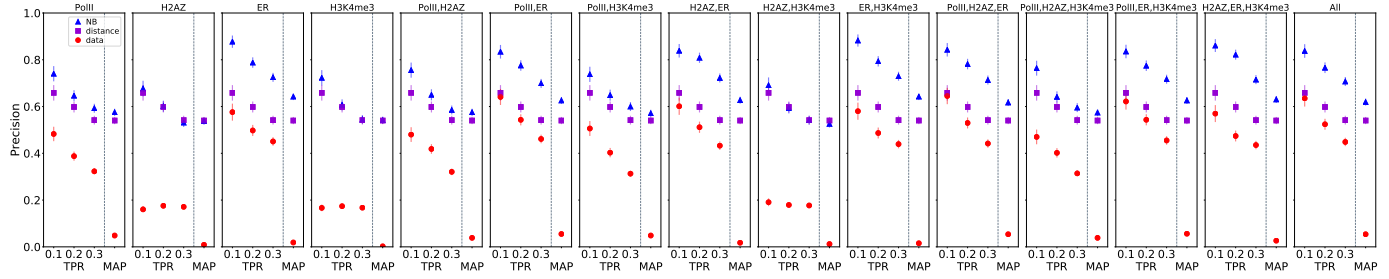

(b) intra-domain performance

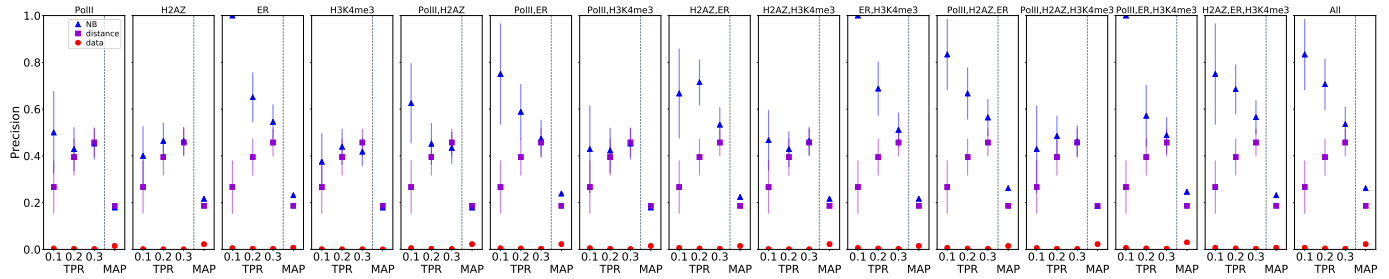

(c) inter-domain performance

Supplement: Figure S7 — Figure shows the comparison of performance of the NB model on odd chromosomes (training data) measured by Precision-TPR and MAP scores. The Precision-TPR curves show the accuracy of the predictions with the highest 10%, 20%, 30% scores i.e. posterior probabilities. The second and the third rows stratify predictions at each of the thresholds into those which take place within domains and those involving inter-domain contacts. The set of positive and negative pairs for the first model was constructed using 1500bp-upstream- extended-genes and distal enhancers. The correlation-based attributes of the two models were estimated using signals (time series) aggregated over 300bp- upstream-extended-genes, and distal enhancer bodies. The separation-based feature was estimated from 1500bp-upstream-shifted TSS to the centres of the ER-α enhancers. [file peerj-05-3742-s007.pdf]

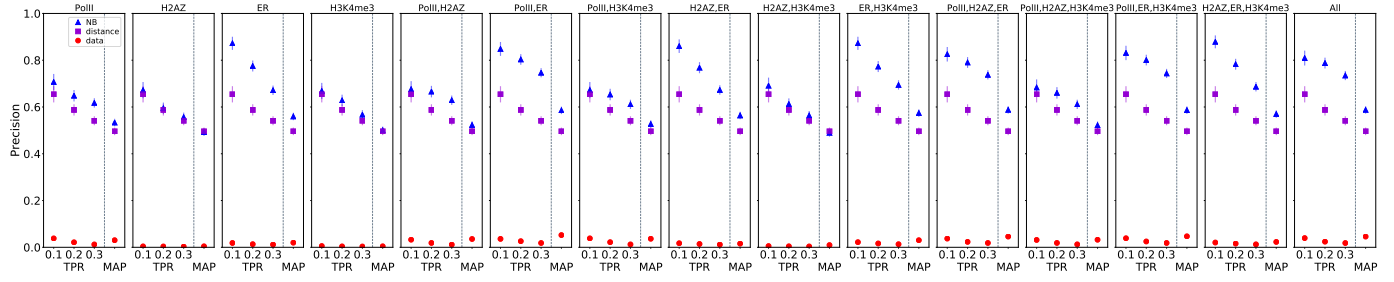

(a) test data performance

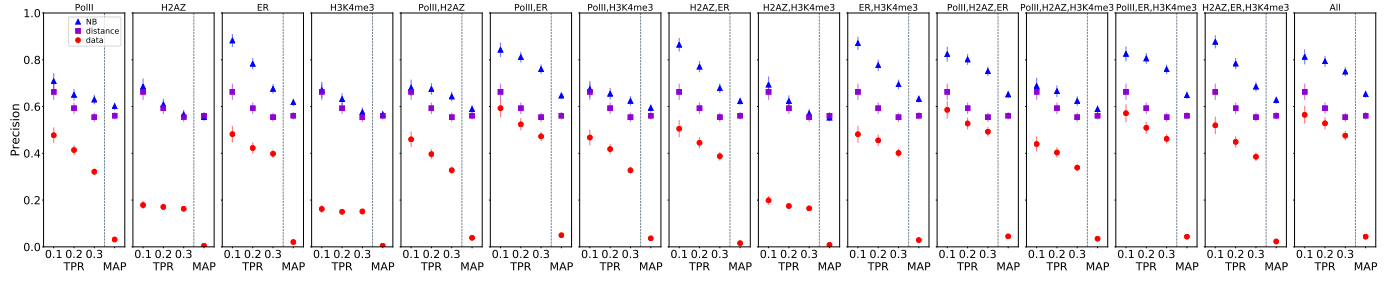

(b) intra-domain performance

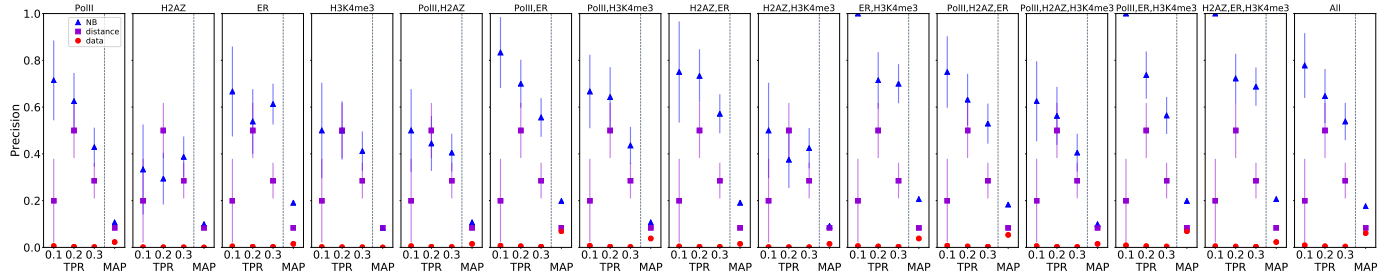

(c) inter-domain performance

Supplement: Figure S8 — Figure shows the comparison of performance of the NB model on even chromosomes (test data) measured by Precision-TPR and MAP scores. The Precision-TPR curves show the accuracy of the predictions with the highest 10%, 20%, 30% scores i.e. posterior probabilities. The second and the third rows stratify predictions at each of the thresholds into those which take place within domains and those involving inter-domain contacts. The set of positive and negative pairs for the first model was constructed using 1500bp-upstream- extended-genes and distal enhancers. The correlation-based attributes of the two models were estimated using signals (time series) aggregated over 300bp- upstream-extended-genes, and distal enhancer bodies. The separation-based feature was estimated from 1,500 bp-upstream-shifted TSS to the centres of the ER-α enhancers. [file peerj-05-3742-s008.pdf]

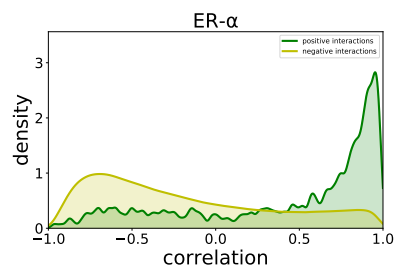

(a)

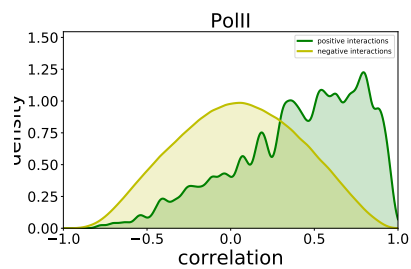

(b)

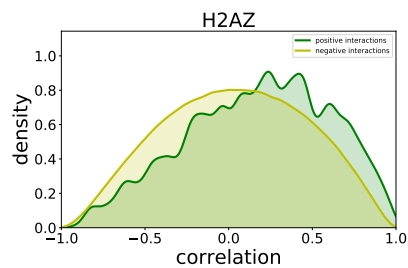

(c)

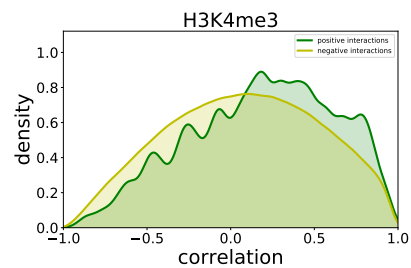

(d)

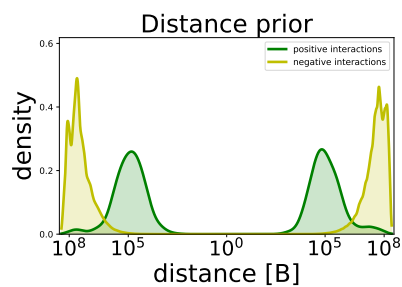

(e)

Supplement: Figure S11 — The graphs (a, b, c, d) show positive (green) and negative (yellow) distributions of correlations between time series of 300 bp-upstream-extended- gene regions and enhancer bodies (MACS: λ local on, p-value 10e−05) for ER-α, PolII, H2AZ and H3K4me3 collected across all odd chromosomes. The figure (e) shows the distribution of genomic distances between centres of distal enhancers and 300 p-upstream-shifted-TSS of genes. The set of positive and negative pairs was constructed using 300 p-upstream-extended-genes and distal enhancers. [file peerj-05-3742-s011.pdf]

Overall performance

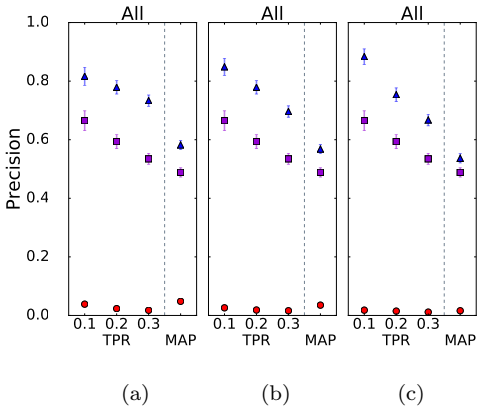

Supplement: Figure S12 — Figure shows the comparison of performance of the NB model on even chromosomes (test data) with correlation-based features calculated using the first (a) 8, (b) 6, (c) 4 time points of our logarithmically spaced time course data. The performance was measured using Precision-TPR and MAP scores. The Precision-TPR curves show the accuracy of the predictions with the highest 10%, 20%, 30% scores i.e., posterior probabilities. The set of positive and negative pairs for the first model was constructed using 300 bp-upstream-extended-genes and distal enhancers. The correlation-based attributes of the two models were estimated using signals (time series) aggregated over 300 bp-upstream-extended-genes, and distal enhancer bodies. The separation-based feature was estimated from 300 bp-upstream-shifted TSS to the centres of the ER-α enhancers. [file peerj-05-3742-s012.pdf]
