# Supplementary material for: Predicting stimulation-dependent enhancer-promoter interactions from ChIP-Seq time course data
Source: PeerJ. 2017 Sep 28;5:e3742. doi: 10.7717/peerj.3742 (PMC5623311; doi:10.7717/peerj.3742)
Supplement: Figure S9 — The first column of the figure shows the performance of the NB model on all odd chromosomes. The model was trained on the stringent time persistent merged MACS-called peaks (i.e., distal ER-α bindings) from the scan with the p- value of 1e−07 and the local control switched off, in which case the search is done with λ BG . In the second column we see the performance under the alternative peak calling with the p-value of 1e−05 (MACS’ default), no control and the local control flag on. The set of positive and negative pairs for the first model was constructed using 300 bp-upstream-extended-genes and distal enhancers. The correlation-based attributes of the model were estimated using pairs of 300 bp- upstream-extended-genes, and enhancers (merged distal MACS-called peaks). The separation-based feature was estimated from 300bp-upstream-shifted TSS to the centres of the ER-α enhancers. [file peerj-05-3742-s009.pdf]

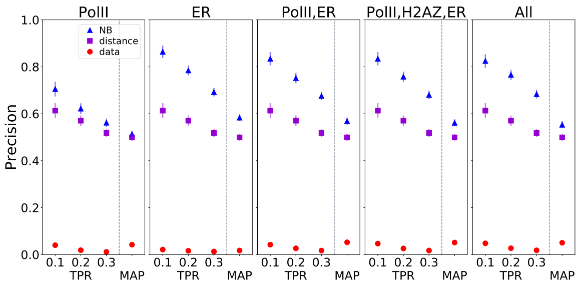

(a) training data performance

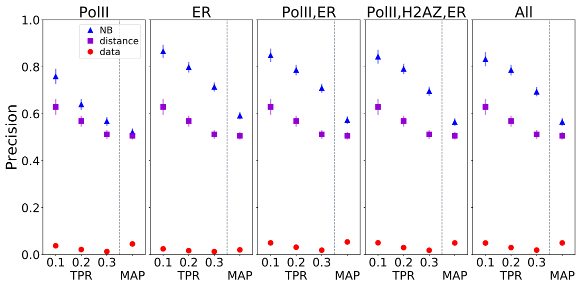

(b) test data performance

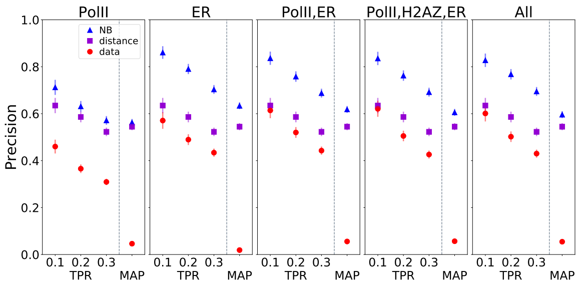

(c) training data intra-genic

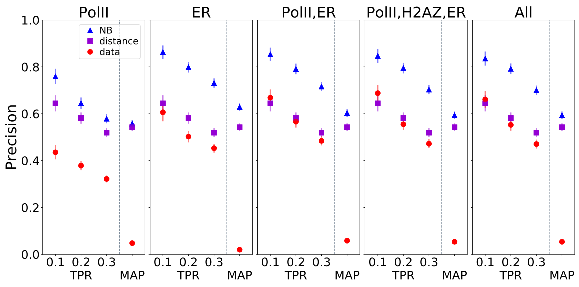

(d) test data intra-genic

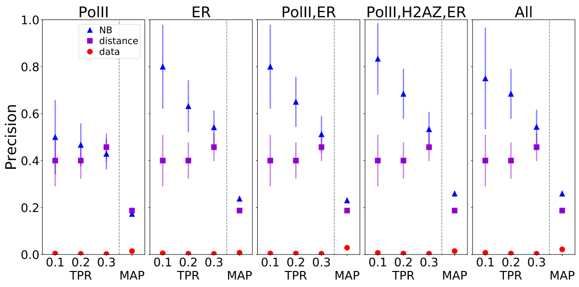

(e) training data inter-genic

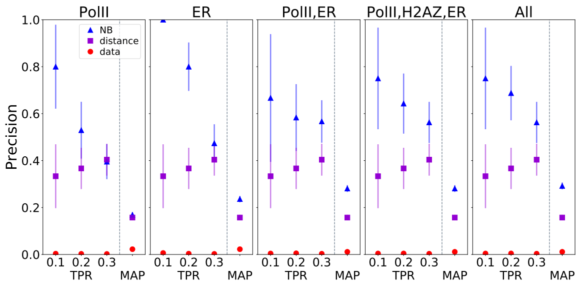

(f) test data inter-genic
