# Supplementary material for: Predicting stimulation-dependent enhancer-promoter interactions from ChIP-Seq time course data
Source: PeerJ. 2017 Sep 28;5:e3742. doi: 10.7717/peerj.3742 (PMC5623311; doi:10.7717/peerj.3742)
Supplement: Table S1 [file peerj-05-3742-s013.doc]

| Gene Name | Distal Enhancers | Proximal Enhancers | NegativeLogLikelihood |
| --- | --- | --- | --- |
| ADAMTS9 | 229 | 135 | 412.62 |
| DOK5 | 152 | 106 | 240.54 |
| PPM1E | 8 | 159 | 9.49 |
| NCOA3 | 31 | 89 | 33.05 |
| PRICKLE2 | 43 | 56 | 58.11 |
| ZMYND8 | 17 | 81 | 20.73 |
| SULF2 | 15 | 74 | 20.25 |
| BCAS3 | 16 | 58 | 18.07 |
| PDE4DIP | 14 | 49 | 16.98 |
| PREX1 | 5 | 56 | 5.23 |
| TRIM37 | 32 | 21 | 51.82 |
| GRHL2 | 25 | 22 | 54.46 |
| BCAS1 | 32 | 15 | 46.69 |
| IGF1R | 2 | 44 | 2.35 |
| TRIM33 | 1 | 45 | 1.07 |
| DSCAM | 1 | 44 | 1.63 |
| NTNG1 | 10 | 34 | 10.63 |
| WWOX | 6 | 37 | 10.57 |
| ZNF217 | 28 | 9 | 46.68 |
| ZFHX3 | 5 | 32 | 13.14 |
| MYC | 34 | 2 | 84.49 |
| TRAPPC9 | 1 | 35 | 1.95 |
| NRIP1 | 31 | 4 | 88.17 |
| TMEM75 | 34 | 1 | 80.92 |
| EYA2 | 1 | 34 | 0.96 |
| CA4 | 23 | 10 | 28.56 |
| VAV3 | 1 | 32 | 1.56 |
| MSI2 | 1 | 32 | 0.89 |
| NAV2 | 2 | 30 | 3.18 |
| FAM65C | 3 | 29 | 3.07 |
| CUX1 | 1 | 30 | 1.29 |
| TFAP2C | 28 | 2 | 45.02 |
| TBX4 | 21 | 9 | 29.82 |
| ATXN7 | 1 | 29 | 1.11 |
| KLF10 | 25 | 4 | 59.49 |
| HES1 | 27 | 2 | 56.97 |
| STARD13 | 19 | 10 | 38.98 |
| 01/03/10 | 22 | 7 | 37.02 |
| ZMIZ1 | 5 | 24 | 10.8 |
| DIP2C | 3 | 26 | 7.96 |
| ITPK1 | 3 | 26 | 5.59 |
| THSD4 | 2 | 27 | 3.5 |
| PRKAG2 | 2 | 27 | 3.42 |
| NCOR2 | 10 | 18 | 14.56 |
| SLC24A3 | 5 | 23 | 7.91 |
| LDLRAD4 | 1 | 27 | 0.85 |
| TOB1 | 22 | 4 | 48.09 |
| PMEPA1 | 15 | 11 | 27.14 |
| INPP5A | 7 | 19 | 11.64 |
| C9orf3 | 7 | 19 | 8.13 |
| VMP1 | 1 | 25 | 0.87 |
| ACKR3 | 23 | 2 | 36.89 |
| NACA2 | 24 | 1 | 30.45 |
| PITPNC1 | 1 | 24 | 1.53 |
| BAMBI | 22 | 2 | 33.91 |
| MECOM | 20 | 4 | 31.21 |
| PARD6B | 16 | 8 | 24.31 |
| C1orf21 | 5 | 19 | 14.74 |
| GSE1 | 9 | 15 | 12.86 |
| CADPS | 6 | 18 | 7.82 |
| BMP7 | 2 | 22 | 3.32 |
| HIVEP3 | 2 | 22 | 3.02 |
| GNA14 | 5 | 18 | 14.5 |
| EVL | 4 | 19 | 5.81 |
| RP11-15E18.4 | 4 | 19 | 4.5 |
| RXRA | 2 | 21 | 2.29 |
| CMIP | 2 | 21 | 2.06 |
| ATP9A | 1 | 22 | 1.41 |
| PRKCA | 1 | 22 | 1.12 |
| LRP1B | 1 | 22 | 1.05 |
| B3GALNT1 | 20 | 2 | 32.38 |
| GREB1 | 11 | 11 | 20.35 |
| RASEF | 11 | 11 | 20.14 |
| TLE1 | 10 | 12 | 13.37 |
| ELMSAN1 | 2 | 20 | 2.71 |
| TTC7A | 1 | 21 | 0.79 |
| SDC1 | 15 | 6 | 30.81 |
| TBX2 | 16 | 5 | 25.58 |
| RREB1 | 14 | 7 | 19.46 |
| CBFA2T3 | 13 | 8 | 19.14 |
| RAB31 | 8 | 13 | 12.15 |
| SLC6A6 | 7 | 14 | 11.76 |
| SPATA13 | 2 | 19 | 5.29 |
| CLMN | 2 | 19 | 3.23 |
| TSNARE1 | 2 | 19 | 3.09 |
| ARSG | 2 | 19 | 3.02 |
| CDH4 | 3 | 18 | 2.87 |
| CTNND2 | 1 | 20 | 1.24 |
| ANKRD11 | 1 | 20 | 0.97 |
| FAM174B | 11 | 9 | 30.82 |
| TLE3 | 17 | 3 | 26.73 |
| METRNL | 14 | 6 | 24.28 |
| SVIL | 8 | 12 | 18.07 |
| BMPER | 7 | 13 | 11.17 |
| COL18A1 | 5 | 15 | 8.16 |
| KSR1 | 5 | 15 | 6.06 |
| SHB | 3 | 17 | 3.82 |
| ATXN7L1 | 1 | 19 | 1.23 |
| ANO6 | 1 | 19 | 0.82 |
| SIAH2 | 13 | 6 | 36.67 |
| NR5A2 | 7 | 12 | 14.34 |
| RBM47 | 5 | 14 | 9.2 |
| KRT8 | 6 | 13 | 7.41 |
| ABHD2 | 4 | 15 | 6.73 |
| MPPED2 | 3 | 16 | 5.65 |
| WWC1 | 1 | 18 | 2.78 |
| MAGI1 | 1 | 18 | 1.79 |
| SIK1 | 16 | 2 | 33.18 |
| KLF4 | 17 | 1 | 32.72 |
| EFNA5 | 7 | 11 | 15.22 |
| CDH18 | 5 | 13 | 11.6 |
| RASSF3 | 4 | 14 | 9.91 |
| KCNMA1 | 4 | 14 | 6.12 |
| DAPK1 | 4 | 14 | 5.43 |
| UST | 3 | 15 | 4.74 |
| TANC1 | 3 | 15 | 4 |
| ITGB5 | 3 | 15 | 3.76 |
| KCND3 | 2 | 16 | 2.74 |
| RAD51C | 2 | 16 | 1.96 |
| CTBP2 | 2 | 16 | 1.77 |
| TENM4 | 1 | 17 | 1.55 |
| PLEKHA7 | 1 | 17 | 1.34 |
| ARHGAP10 | 1 | 17 | 1.33 |
| BTBD9 | 1 | 17 | 1.04 |
| PARD3 | 1 | 17 | 0.88 |
| CTC-236F12.4 | 14 | 3 | 35.2 |
| FIGN | 10 | 7 | 24.97 |
| NR2F2 | 15 | 2 | 21.82 |
| SNTN | 14 | 3 | 21.07 |
| RIPK4 | 14 | 3 | 19.28 |
| TPD52L1 | 6 | 11 | 13.94 |
| TGFB2 | 8 | 9 | 13.91 |
| JARID2 | 8 | 9 | 13.86 |
| CYP24A1 | 11 | 6 | 12.48 |
| MREG | 5 | 12 | 11.18 |
| NCKAP5 | 3 | 14 | 6.28 |
| SYNPR | 4 | 13 | 4.3 |
| HEATR6 | 3 | 14 | 3.63 |
| GPR133 | 3 | 14 | 3.12 |
| CDH1 | 3 | 14 | 3.09 |
| DLG2 | 1 | 16 | 1.47 |
| CXXC5 | 9 | 7 | 33.68 |
| KLF6 | 13 | 3 | 30.16 |
| SEMA3C | 8 | 8 | 21.9 |
| FOXO3 | 10 | 6 | 19.76 |
| HK2 | 7 | 9 | 16.24 |
| NRP1 | 7 | 9 | 15.05 |
| SLC38A1 | 7 | 9 | 13.04 |
| TP63 | 6 | 10 | 11.67 |
| ZNF462 | 6 | 10 | 8.69 |
| YPEL2 | 5 | 11 | 8.21 |
| ACTN1 | 4 | 12 | 8.15 |
| BASP1 | 6 | 10 | 7.46 |
| DAAM1 | 2 | 14 | 6.69 |
| TMEM164 | 2 | 14 | 5.36 |
| CDYL2 | 3 | 13 | 4.97 |
| PITPNM2 | 1 | 15 | 1.45 |
| EMP2 | 11 | 4 | 22.98 |
| JAK1 | 6 | 9 | 22.24 |
| SPATS2L | 8 | 7 | 20.42 |
| C14orf182 | 8 | 7 | 18.96 |
| ELF3 | 12 | 3 | 15.91 |
| ATP13A3 | 11 | 4 | 15.73 |
| ARFGEF2 | 10 | 5 | 15.26 |
| ARRB1 | 6 | 9 | 11.66 |
| RP11-293M10.1 | 7 | 8 | 11.57 |
| TSPAN5 | 5 | 10 | 11.24 |
| ESR1 | 8 | 7 | 10.79 |
| PXDN | 5 | 10 | 9.04 |
| NECAB1 | 5 | 10 | 8.56 |
| ST3GAL1 | 7 | 8 | 7.42 |
| GREB1L | 3 | 12 | 6.78 |
| SMAD3 | 4 | 11 | 6.4 |
| NCAM2 | 3 | 12 | 5.73 |
| CCDC88C | 3 | 12 | 4.93 |
| ATXN1 | 3 | 12 | 3.54 |
| ARID5B | 3 | 12 | 3.49 |
| FARP1 | 2 | 13 | 2.5 |
| PLXNA4 | 2 | 13 | 2.13 |
| CRISPLD2 | 2 | 13 | 2.07 |
| SLC1A2 | 1 | 14 | 1.6 |
| ASTN2 | 1 | 14 | 0.9 |
| PODXL | 12 | 2 | 38.03 |
| UGCG | 13 | 1 | 27.45 |
| CCR7 | 13 | 1 | 23.87 |
| ISOC1 | 13 | 1 | 23.38 |
| IRF2BPL | 12 | 2 | 17.38 |
| LPCAT1 | 6 | 8 | 15.07 |
| TMPRSS2 | 8 | 6 | 12.01 |
| NHSL1 | 5 | 9 | 11.53 |
| CCND1 | 11 | 3 | 11.43 |
| ARID1B | 3 | 11 | 8.13 |
| ARHGAP42 | 3 | 11 | 7.13 |
| PTPN1 | 6 | 8 | 6.51 |
| FAM102A | 6 | 8 | 5.95 |
| B3GALT5 | 4 | 10 | 5.29 |
| SLCO3A1 | 3 | 11 | 4.91 |
| NTRK2 | 3 | 11 | 4.54 |
| ARHGEF10L | 4 | 10 | 4.52 |
| AFF1 | 2 | 12 | 4.51 |
| TRIO | 3 | 11 | 4.3 |
| ARAP1 | 2 | 12 | 3.42 |
| FLNB | 2 | 12 | 3.15 |
| RBPMS | 2 | 12 | 2.64 |
| INPP4B | 2 | 12 | 1.91 |
| IGSF3 | 2 | 12 | 1.75 |
| ANK3 | 1 | 13 | 1.51 |
| CELSR1 | 1 | 13 | 1.49 |
| ME3 | 1 | 13 | 1.24 |
| TPRG1 | 1 | 13 | 1.1 |
| FGFR2 | 1 | 13 | 1.01 |
| LPP | 1 | 13 | 0.98 |
| GADD45G | 11 | 2 | 19.78 |
| MYB | 10 | 3 | 19.72 |
| AXIN2 | 10 | 3 | 17.35 |
| ABHD17C | 6 | 7 | 16.7 |
| KCNK5 | 9 | 4 | 12.46 |
| WWC3 | 4 | 9 | 11.58 |
| TRPS1 | 8 | 5 | 11.13 |
| STC2 | 9 | 4 | 10.85 |
| CACNG4 | 9 | 4 | 10.31 |
| PLAC1 | 6 | 7 | 9.78 |
| CASQ2 | 5 | 8 | 9.06 |
| RAPGEF1 | 4 | 9 | 8.18 |
| ESRRG | 6 | 7 | 7.7 |
| C6orf132 | 5 | 8 | 7.23 |
| TEX36 | 3 | 10 | 7.16 |
| RGS9 | 5 | 8 | 6.74 |
| NBPF1 | 4 | 9 | 6.74 |
| FSIP1 | 3 | 10 | 6.66 |
| CREB3L2 | 4 | 9 | 5.41 |
| SLC7A5 | 5 | 8 | 4.96 |
| UXS1 | 2 | 11 | 4.61 |
| MLPH | 3 | 10 | 4.34 |
| PACS2 | 4 | 9 | 3.88 |
| IFT122 | 2 | 11 | 3.57 |
| NTN1 | 2 | 11 | 2.64 |
| PAK4 | 1 | 12 | 2.4 |
| NFATC2 | 2 | 11 | 2.28 |
| DAB2IP | 1 | 12 | 2.19 |
| LRIG1 | 1 | 12 | 1.94 |
| PACS1 | 1 | 12 | 1.43 |
| CAMK1D | 1 | 12 | 1.22 |
| GABBR2 | 1 | 12 | 1.02 |
| SRGAP3 | 1 | 12 | 0.98 |
| CAPZB | 1 | 12 | 0.94 |
| RAB20 | 9 | 3 | 30.1 |
| NXNL2 | 10 | 2 | 25.62 |
| FAM84B | 10 | 2 | 20 |
| DMRT2 | 10 | 2 | 18.18 |
| AGR3 | 10 | 2 | 16.29 |
| TPTE | 12 | 0 | 16.12 |
| CLDN4 | 8 | 4 | 13.47 |
| KCNK1 | 7 | 5 | 12.23 |
| RAB11FIP1 | 7 | 5 | 11.96 |
| CAND1 | 10 | 2 | 10.88 |
| FHL2 | 5 | 7 | 10.06 |
| PIP4K2A | 3 | 9 | 7.79 |
| ZSWIM6 | 6 | 6 | 7.74 |
| C3orf14 | 6 | 6 | 7.55 |
| SCARB1 | 7 | 5 | 7.06 |
| PTPRE | 3 | 9 | 5.89 |
| RAB30 | 4 | 8 | 5.8 |
| TNFAIP8L3 | 4 | 8 | 5.43 |
| PKP1 | 3 | 9 | 4.29 |
| DAPK2 | 2 | 10 | 4 |
| MBOAT1 | 3 | 9 | 3.56 |
| HK1 | 3 | 9 | 3.49 |
| ARHGAP26 | 2 | 10 | 2.54 |
| HPCAL1 | 2 | 10 | 2.51 |
| CTPS2 | 2 | 10 | 2.39 |
| USP31 | 2 | 10 | 2.21 |
| ABCC1 | 2 | 10 | 2.18 |
| TMTC2 | 1 | 11 | 1.68 |
| PDE4D | 1 | 11 | 1.47 |
| FAM129B | 1 | 11 | 1.16 |
| ABLIM2 | 1 | 11 | 0.95 |
| LRCH1 | 1 | 11 | 0.84 |
| NFKBIA | 10 | 1 | 19.08 |
| ALDH1A3 | 8 | 3 | 18.04 |
| DNAJB6 | 9 | 2 | 12.82 |
| IER5 | 9 | 2 | 12.77 |
| PGR | 8 | 3 | 12.04 |
| PABPC1 | 9 | 2 | 12.01 |
| PRSS23 | 7 | 4 | 11.83 |
| SH3YL1 | 8 | 3 | 11.78 |
| FREM2 | 6 | 5 | 10.42 |
| MTUS1 | 4 | 7 | 10.18 |
| PPARG | 5 | 6 | 9.9 |
| FAM83B | 5 | 6 | 9.55 |
| RND3 | 8 | 3 | 9.52 |
| LCMT1 | 6 | 5 | 9.07 |
| NT5C2 | 4 | 7 | 7.98 |
| PFKP | 7 | 4 | 7.92 |
| ETNK2 | 6 | 5 | 7.48 |
| RCL1 | 6 | 5 | 7.43 |
| DOPEY2 | 5 | 6 | 7.09 |
| BDH1 | 5 | 6 | 7.03 |
| CELSR2 | 5 | 6 | 6.86 |
| MKL2 | 4 | 7 | 6.78 |
| AR | 3 | 8 | 6.08 |
| TTC39B | 5 | 6 | 5.77 |
| AGO2 | 2 | 9 | 5.07 |
| LIPC | 4 | 7 | 4.81 |
| NDRG1 | 3 | 8 | 4.29 |
| SGK1 | 4 | 7 | 4.07 |
| MB21D2 | 1 | 10 | 4.07 |
| SYT6 | 4 | 7 | 3.87 |
| CD9 | 3 | 8 | 3.79 |
| WWP1 | 3 | 8 | 3.52 |
| GPC6 | 1 | 10 | 2.44 |
| ST7 | 2 | 9 | 2.01 |
| DGKZ | 1 | 10 | 1.09 |
| TBC1D9 | 1 | 10 | 1.06 |
| USP10 | 1 | 10 | 0.93 |
| MYO3B | 1 | 10 | 0.8 |
| SMAD7 | 9 | 1 | 22.45 |
| FOSL2 | 9 | 1 | 14.73 |
| RHOU | 8 | 2 | 13.32 |
| GPR37L1 | 8 | 2 | 11.33 |
| PFKFB3 | 6 | 4 | 11.32 |
| GATA3 | 6 | 4 | 10.47 |
| CRADD | 5 | 5 | 9.41 |
| BATF | 6 | 4 | 8.39 |
| RFTN1 | 5 | 5 | 8.03 |
| RET | 4 | 6 | 7.77 |
| ARHGEF26 | 2 | 8 | 7.39 |
| PSMD6 | 7 | 3 | 7.25 |
| ATP8B1 | 4 | 6 | 6.67 |
| UBE3C | 4 | 6 | 6.31 |
| BACE2 | 5 | 5 | 5.8 |
| ZBTB40 | 4 | 6 | 5.26 |
| SH3BP4 | 3 | 7 | 4.41 |
| ZMYND11 | 3 | 7 | 4.39 |
| STON2 | 3 | 7 | 4.37 |
| NFIB | 3 | 7 | 4.14 |
| SASH1 | 3 | 7 | 3.98 |
| NADSYN1 | 3 | 7 | 3.76 |
| PRKCE | 3 | 7 | 3.5 |
| MBNL2 | 2 | 8 | 3.31 |
| NAALADL2 | 2 | 8 | 3.21 |
| CDH26 | 3 | 7 | 3.04 |
| YWHAZ | 3 | 7 | 3.02 |
| SLC25A25 | 3 | 7 | 2.88 |
| GLTSCR1 | 2 | 8 | 2.87 |
| LMX1B | 2 | 8 | 2.75 |
| CCDC6 | 2 | 8 | 2.71 |
| KAT6B | 2 | 8 | 2.63 |
| CORO2A | 2 | 8 | 2.31 |
| SETBP1 | 2 | 8 | 1.93 |
| NYAP2 | 2 | 8 | 1.8 |
| SYNJ2 | 1 | 9 | 1.65 |
| KIF16B | 1 | 9 | 1.39 |
| COL27A1 | 1 | 9 | 1.29 |
| PLCB1 | 1 | 9 | 1.2 |
| SLC27A2 | 1 | 9 | 1.19 |
| SULT2B1 | 1 | 9 | 1.1 |
| SH3BP5 | 1 | 9 | 1.05 |
| SH3RF2 | 1 | 9 | 1.05 |
| TAF3 | 1 | 9 | 1.02 |
| TLN2 | 1 | 9 | 1 |
| DENND5B | 1 | 9 | 1 |
| CCDC85C | 1 | 9 | 0.99 |
| TMEM120B | 1 | 9 | 0.91 |
| ZPLD1 | 1 | 9 | 0.88 |
| RMI2 | 1 | 9 | 0.84 |
| PTK2 | 1 | 9 | 0.81 |
| LRP5 | 1 | 9 | 0.8 |
| FAM178B | 1 | 9 | 0.8 |
| CLSTN2 | 1 | 9 | 0.79 |
| P2RY2 | 6 | 3 | 18.9 |
| N4BP3 | 6 | 3 | 16.5 |
| BCOR | 8 | 1 | 15.42 |
| SMOX | 5 | 4 | 15.39 |
| FAM189A2 | 4 | 5 | 14.22 |
| MEIS1 | 6 | 3 | 14.1 |
| DUSP4 | 9 | 0 | 13.41 |
| OPHN1 | 4 | 5 | 13.11 |
| OBFC1 | 7 | 2 | 12.69 |
| MCCC2 | 6 | 3 | 12.68 |
| RP11-1070N10.3 | 8 | 1 | 12.44 |
| SLC29A1 | 8 | 1 | 11.88 |
| TSPAN15 | 6 | 3 | 11.79 |
| INHBB | 8 | 1 | 11.55 |
| CD47 | 8 | 1 | 11.17 |
| CDKL4 | 6 | 3 | 11.16 |
| MPHOSPH6 | 7 | 2 | 11.14 |
| FAT4 | 6 | 3 | 11.11 |
| COX7A2L | 5 | 4 | 11 |
| RDX | 6 | 3 | 10.49 |
| C9orf85 | 7 | 2 | 10.18 |
| CPT1A | 5 | 4 | 8.9 |
| CDH17 | 7 | 2 | 8.83 |
| ADCY5 | 4 | 5 | 8.5 |
| IP6K3 | 4 | 5 | 8.32 |
| KRT19 | 5 | 4 | 8.31 |
| ERICH1 | 3 | 6 | 8.07 |
| CAP2 | 3 | 6 | 8.04 |
| RERG | 4 | 5 | 6.98 |
| S100A10 | 4 | 5 | 5.7 |
| CLIC6 | 4 | 5 | 5.47 |
| TNFRSF19 | 2 | 7 | 5.12 |
| CHSY1 | 4 | 5 | 4.95 |
| STK39 | 2 | 7 | 4.9 |
| ATP7B | 4 | 5 | 4.89 |
| SBNO2 | 4 | 5 | 4.65 |
| PDPR | 3 | 6 | 4.33 |
| CSRP1 | 4 | 5 | 4.33 |
| ANGPT1 | 4 | 5 | 4.13 |
| PTPRK | 3 | 6 | 3.7 |
| CERS6 | 2 | 7 | 3.37 |
| ERBB4 | 2 | 7 | 3.33 |
| CHST15 | 3 | 6 | 3.02 |
| MICAL2 | 2 | 7 | 2.99 |
| LAMC1 | 2 | 7 | 2.97 |
| BCMO1 | 3 | 6 | 2.95 |
| SCIN | 2 | 7 | 2.94 |
| 01/03/14 | 2 | 7 | 2.84 |
| MPRIP | 2 | 7 | 2.59 |
| TANC2 | 2 | 7 | 2.39 |
| POR | 2 | 7 | 2.25 |
| SNTB1 | 2 | 7 | 2.19 |
| ATP6V1C2 | 1 | 8 | 2.11 |
| GALNT2 | 2 | 7 | 2.1 |
| MITF | 1 | 8 | 2.01 |
| CACNA1I | 1 | 8 | 1.64 |
| IPPK | 1 | 8 | 1.56 |
| SLCO2A1 | 1 | 8 | 1.44 |
| LYPD6B | 1 | 8 | 1.31 |
| PRMT8 | 1 | 8 | 1.29 |
| WNK2 | 1 | 8 | 1.02 |
| CA10 | 1 | 8 | 0.96 |
| ENOX1 | 1 | 8 | 0.87 |
| PPM1H | 1 | 8 | 0.85 |
| HSPB8 | 6 | 2 | 18.7 |
| C1QTNF6 | 7 | 1 | 18.48 |
| PBX1 | 8 | 0 | 16.62 |
| ACTBL2 | 8 | 0 | 14.03 |
| FXYD4 | 6 | 2 | 13.22 |
| C6orf141 | 8 | 0 | 12.51 |
| P2RX2 | 7 | 1 | 11.4 |
| IRF2BP2 | 7 | 1 | 11.18 |
| OSR2 | 6 | 2 | 10.97 |
| ALOX5AP | 6 | 2 | 10.88 |
| GOLPH3 | 5 | 3 | 10.85 |
| LYZL1 | 7 | 1 | 10.82 |
| TPBG | 6 | 2 | 10.3 |
| C2orf54 | 6 | 2 | 9.97 |
| AL031666.2 | 7 | 1 | 9.55 |
| LY6E | 6 | 2 | 9.22 |
| TSHZ3 | 5 | 3 | 9.11 |
| POU1F1 | 6 | 2 | 9.09 |
| NFATC1 | 4 | 4 | 8.61 |
| NUDT7 | 7 | 1 | 8.22 |
| ABLIM1 | 5 | 3 | 8.11 |
| MAP3K8 | 5 | 3 | 7.91 |
| HDAC11 | 7 | 1 | 7.45 |
| FAM134B | 6 | 2 | 7.28 |
| SCUBE2 | 2 | 6 | 6.63 |
| ASB13 | 3 | 5 | 6.63 |
| AMOTL2 | 4 | 4 | 6.37 |
| GTF3A | 6 | 2 | 6.12 |
| KIAA1467 | 4 | 4 | 6.12 |
| HAAO | 3 | 5 | 6.05 |
| EPAS1 | 4 | 4 | 5.53 |
| NEDD9 | 5 | 3 | 5.48 |
| KCTD3 | 5 | 3 | 5.33 |
| PDLIM1 | 3 | 5 | 5.22 |
| CD59 | 4 | 4 | 5.16 |
| P2RX7 | 3 | 5 | 4.49 |
| PPP2R2A | 4 | 4 | 4.29 |
| SLC25A45 | 4 | 4 | 4.14 |
| DOK7 | 2 | 6 | 4.13 |
| LURAP1L | 2 | 6 | 3.98 |
| MAFK | 3 | 5 | 3.87 |
| TP53INP1 | 4 | 4 | 3.86 |
| KIF21A | 1 | 7 | 3.77 |
| ANXA3 | 2 | 6 | 3.72 |
| EXT1 | 2 | 6 | 3.66 |
| SRD5A3 | 3 | 5 | 3.53 |
| FBP1 | 3 | 5 | 3.51 |
| SERPINA6 | 3 | 5 | 3.5 |
| SEPT9 | 2 | 6 | 3.41 |
| SLC13A2 | 3 | 5 | 3.36 |
| SPIRE2 | 2 | 6 | 2.99 |
| LRRC1 | 2 | 6 | 2.89 |
| SMURF2 | 3 | 5 | 2.86 |
| ACSS1 | 2 | 6 | 2.85 |
| FAM83A | 3 | 5 | 2.71 |
| CACNA2D4 | 1 | 7 | 2.44 |
| SEMA4B | 2 | 6 | 2.41 |
| SEMA4D | 2 | 6 | 2.41 |
| BAIAP2L1 | 1 | 7 | 2.37 |
| ESR2 | 1 | 7 | 2.33 |
| GPR157 | 2 | 6 | 2.16 |
| LRFN5 | 2 | 6 | 1.97 |
| GTF2IRD1 | 2 | 6 | 1.9 |
| WNT9A | 1 | 7 | 1.77 |
| SPSB1 | 1 | 7 | 1.63 |
| MARK2 | 1 | 7 | 1.46 |
| RADIL | 1 | 7 | 1.39 |
| THRB | 1 | 7 | 1.3 |
| ANKRD29 | 1 | 7 | 1.2 |
| EEPD1 | 1 | 7 | 1.19 |
| SMPD3 | 1 | 7 | 1.17 |
| TTC21A | 1 | 7 | 1.17 |
| FAM53B | 1 | 7 | 1.07 |
| AMZ1 | 1 | 7 | 1.06 |
| SLC44A1 | 1 | 7 | 1 |
| CGNL1 | 1 | 7 | 0.95 |
| FGD6 | 1 | 7 | 0.93 |
| KDM4B | 1 | 7 | 0.92 |
| VGLL4 | 1 | 7 | 0.84 |
| KLF9 | 6 | 1 | 27.61 |
| CYP1B1 | 6 | 1 | 16.64 |
| TMSB4X | 7 | 0 | 15.14 |
| HSPB1 | 6 | 1 | 14.1 |
| ULK1 | 7 | 0 | 13.44 |
| TSKU | 5 | 2 | 13.15 |
| AL133318.1 | 6 | 1 | 12.22 |
| EREG | 6 | 1 | 10.82 |
| AC016885.1 | 7 | 0 | 10.75 |
| DHRS3 | 4 | 3 | 10.1 |
| MAL | 3 | 4 | 9.92 |
| LMCD1 | 5 | 2 | 9.2 |
| SMS | 4 | 3 | 8.89 |
| MAPK8 | 5 | 2 | 8.61 |
| TTC6 | 6 | 1 | 8.55 |
| PAPSS2 | 3 | 4 | 8.25 |
| PCYOX1 | 6 | 1 | 7.51 |
| TMEM26 | 5 | 2 | 7.44 |
| MSX2 | 7 | 0 | 7.4 |
| UBL3 | 5 | 2 | 7.23 |
| CAMK2G | 4 | 3 | 6.64 |
| TBCA | 3 | 4 | 6.5 |
| TMCC1 | 3 | 4 | 6.46 |
| TJP1 | 3 | 4 | 6.42 |
| AKAP1 | 5 | 2 | 6.22 |
| ETS2 | 4 | 3 | 6.18 |
| PFDN4 | 3 | 4 | 6.15 |
| TRAF3IP2 | 3 | 4 | 6.05 |
| ICOSLG | 4 | 3 | 5.88 |
| MAB21L3 | 4 | 3 | 5.87 |
| PKD2L2 | 4 | 3 | 5.55 |
| DUSP16 | 2 | 5 | 5.46 |
| PDCD6IP | 3 | 4 | 5.34 |
| FMN1 | 2 | 5 | 4.95 |
| DOCK5 | 2 | 5 | 4.83 |
| DSP | 4 | 3 | 4.75 |
| UBE2I | 5 | 2 | 4.71 |
| FOXA1 | 4 | 3 | 4.71 |
| FAM110B | 3 | 4 | 4.61 |
| IL4R | 3 | 4 | 4.55 |
| PYGL | 3 | 4 | 4.44 |
| ZFAT | 4 | 3 | 4.39 |
| PGS1 | 2 | 5 | 4.13 |
| TGM2 | 3 | 4 | 3.95 |
| ABCG2 | 1 | 6 | 3.79 |
| NPSR1 | 3 | 4 | 3.68 |
| TANK | 3 | 4 | 3.53 |
| ZNF823 | 3 | 4 | 3.43 |
| FOXK1 | 1 | 6 | 3.38 |
| CAPN2 | 2 | 5 | 3.19 |
| TRIM25 | 2 | 5 | 3.08 |
| SH3TC1 | 2 | 5 | 3.06 |
| SNX10 | 3 | 4 | 2.86 |
| WIPI1 | 2 | 5 | 2.77 |
| ACSL3 | 2 | 5 | 2.75 |
| WDR64 | 2 | 5 | 2.74 |
| QSOX1 | 1 | 6 | 2.66 |
| FAM169B | 2 | 5 | 2.65 |
| CADM1 | 2 | 5 | 2.3 |
| TMEM2 | 2 | 5 | 2.18 |
| PLEKHM3 | 1 | 6 | 2.17 |
| ALPK3 | 2 | 5 | 2.16 |
| WIPF1 | 2 | 5 | 2.08 |
| SCUBE1 | 2 | 5 | 1.9 |
| FBXO34 | 1 | 6 | 1.83 |
| MAGI3 | 1 | 6 | 1.82 |
| RAC1 | 2 | 5 | 1.79 |
| FN1 | 1 | 6 | 1.78 |
| PDXDC1 | 1 | 6 | 1.68 |
| POC1B | 2 | 5 | 1.66 |
| NKAIN1 | 1 | 6 | 1.65 |
| TBC1D5 | 1 | 6 | 1.64 |
| SHANK2 | 1 | 6 | 1.39 |
| CDC42BPB | 1 | 6 | 1.25 |
| SLC25A24 | 1 | 6 | 1.24 |
| GSG1L | 1 | 6 | 1.17 |
| MED13L | 1 | 6 | 1.16 |
| CTIF | 1 | 6 | 1.13 |
| PPFIBP1 | 1 | 6 | 1.12 |
| FRMD4B | 1 | 6 | 1.08 |
| SLC9A8 | 1 | 6 | 1.08 |
| CNBD1 | 1 | 6 | 1.05 |
| GALNT7 | 1 | 6 | 0.95 |
| RGS12 | 1 | 6 | 0.95 |
| SH3BP2 | 1 | 6 | 0.94 |
| ZNF827 | 1 | 6 | 0.93 |
| BCL7A | 1 | 6 | 0.88 |
| OXR1 | 1 | 6 | 0.87 |
| RNF144B | 1 | 6 | 0.87 |
| SYTL2 | 1 | 6 | 0.82 |
| FAXDC2 | 1 | 6 | 0.81 |
| ATP6V1F | 5 | 1 | 11.68 |
| AL121761.2 | 6 | 0 | 11.42 |
| SPTSSB | 5 | 1 | 8.75 |
| CA12 | 3 | 3 | 8.22 |
| EDN1 | 6 | 0 | 8 |
| CXorf40B | 5 | 1 | 7.37 |
| AHCY | 5 | 1 | 7.31 |
| RP11-680G10.1 | 5 | 1 | 7.13 |
| SGK223 | 5 | 1 | 7.08 |
| MYLIP | 5 | 1 | 6.97 |
| FZD1 | 5 | 1 | 6.89 |
| SUCLA2 | 4 | 2 | 6.88 |
| CDKN2B | 6 | 0 | 6.83 |
| TFAP2A | 5 | 1 | 6.72 |
| SLC25A36 | 3 | 3 | 6.06 |
| SPOPL | 4 | 2 | 6.04 |
| UGDH | 3 | 3 | 6.03 |
| IGFBP5 | 5 | 1 | 5.65 |
| SNAP29 | 2 | 4 | 5.4 |
| PRICKLE1 | 4 | 2 | 5.35 |
| BTD | 3 | 3 | 5.18 |
| MAOA | 2 | 4 | 4.99 |
| SPPL2A | 4 | 2 | 4.99 |
| BAG3 | 3 | 3 | 4.97 |
| RNF14 | 4 | 2 | 4.97 |
| ANKRD18A | 4 | 2 | 4.89 |
| DDX47 | 4 | 2 | 4.87 |
| TGFBI | 3 | 3 | 4.82 |
| SERPINB9 | 4 | 2 | 4.77 |
| DNAJC5B | 2 | 4 | 4.66 |
| C20orf197 | 2 | 4 | 4.61 |
| FBXO32 | 4 | 2 | 4.61 |
| SULT1C2 | 3 | 3 | 4.57 |
| CHD9 | 1 | 5 | 4.49 |
| GLRX | 3 | 3 | 4.37 |
| TBL1XR1 | 2 | 4 | 4.14 |
| DSCR3 | 2 | 4 | 4.02 |
| ROPN1L | 2 | 4 | 4 |
| SYNGR1 | 4 | 2 | 3.95 |
| USP3 | 3 | 3 | 3.82 |
| PLA2G16 | 2 | 4 | 3.71 |
| BCAR1 | 3 | 3 | 3.64 |
| FAM46A | 3 | 3 | 3.63 |
| ADCY1 | 2 | 4 | 3.63 |
| AC018816.3 | 2 | 4 | 3.58 |
| RANBP9 | 3 | 3 | 3.23 |
| ACSF3 | 3 | 3 | 3.21 |
| CPEB4 | 3 | 3 | 3.13 |
| DIRC3 | 3 | 3 | 3.11 |
| VWA3B | 3 | 3 | 2.98 |
| GOLM1 | 3 | 3 | 2.85 |
| SCOC | 2 | 4 | 2.73 |
| SLC9A4 | 2 | 4 | 2.7 |
| LOXL1 | 2 | 4 | 2.58 |
| GAB2 | 2 | 4 | 2.45 |
| C14orf132 | 2 | 4 | 2.42 |
| B4GALNT3 | 1 | 5 | 2.37 |
| OSTF1 | 1 | 5 | 2.35 |
| NEK6 | 2 | 4 | 2.35 |
| RHOH | 1 | 5 | 2.22 |
| ICA1 | 2 | 4 | 2.11 |
| CIRBP | 2 | 4 | 2.1 |
| NMNAT2 | 2 | 4 | 2.08 |
| BRWD1 | 2 | 4 | 1.97 |
| ERRFI1 | 2 | 4 | 1.93 |
| COLEC12 | 1 | 5 | 1.89 |
| TBC1D8 | 1 | 5 | 1.8 |
| GFPT2 | 2 | 4 | 1.78 |
| MYPN | 2 | 4 | 1.74 |
| CHST12 | 1 | 5 | 1.72 |
| TES | 2 | 4 | 1.67 |
| RAPGEF2 | 1 | 5 | 1.64 |
| NFIC | 1 | 5 | 1.57 |
| OSBPL10 | 1 | 5 | 1.53 |
| NTN4 | 1 | 5 | 1.52 |
| FCGR2B | 1 | 5 | 1.37 |
| SCNN1B | 1 | 5 | 1.34 |
| SLC2A12 | 1 | 5 | 1.32 |
| DAGLA | 1 | 5 | 1.29 |
| JMJD1C | 1 | 5 | 1.25 |
| TGFA | 1 | 5 | 1.25 |
| UMODL1 | 1 | 5 | 1.24 |
| PXN | 1 | 5 | 1.2 |
| MYO6 | 1 | 5 | 1.19 |
| SLC1A1 | 1 | 5 | 1.17 |
| CLIP1 | 1 | 5 | 1.17 |
| EPPK1 | 1 | 5 | 1.14 |
| EHD4 | 1 | 5 | 1.09 |
| CXADR | 1 | 5 | 1.06 |
| DNAH17 | 1 | 5 | 1.04 |
| SLC7A2 | 1 | 5 | 1.01 |
| CHPT1 | 1 | 5 | 1 |
| PDE6A | 1 | 5 | 0.96 |
| ITPKB | 1 | 5 | 0.96 |
| OTUD7B | 1 | 5 | 0.93 |
| PIK3C2B | 1 | 5 | 0.93 |
| LAMB1 | 1 | 5 | 0.93 |
| BCAR3 | 1 | 5 | 0.92 |
| MGAT5 | 1 | 5 | 0.91 |
| PPP1R13B | 1 | 5 | 0.91 |
| ZFPM1 | 1 | 5 | 0.9 |
| MED13 | 1 | 5 | 0.88 |
| HDHD1 | 1 | 5 | 0.87 |
| MYO18A | 1 | 5 | 0.86 |
| DNAJC1 | 1 | 5 | 0.86 |
| RHOF | 1 | 5 | 0.85 |
| CACNA1H | 1 | 5 | 0.8 |
| UBTD1 | 1 | 5 | 0.8 |
| MLLT3 | 1 | 5 | 0.79 |
| PSCA | 4 | 1 | 11.18 |
| TSC22D3 | 5 | 0 | 9.57 |
| RP11-404L6.2 | 4 | 1 | 9.51 |
| PRKCD | 2 | 3 | 8.75 |
| SSR3 | 4 | 1 | 8.04 |
| NR4A3 | 4 | 1 | 7.94 |
| SIGLEC15 | 4 | 1 | 7.91 |
| TXN2 | 5 | 0 | 7.87 |
| RCAN1 | 4 | 1 | 7.67 |
| FAM198B | 3 | 2 | 7.56 |
| CITED2 | 5 | 0 | 7.44 |
| IL6ST | 4 | 1 | 7.35 |
| TP53I11 | 3 | 2 | 7.17 |
| PRDM1 | 5 | 0 | 6.67 |
| S100P | 3 | 2 | 6.26 |
| ALG1L | 5 | 0 | 6.2 |
| FZD7 | 5 | 0 | 6.19 |
| LINC00999 | 5 | 0 | 6.07 |
| PODXL2 | 4 | 1 | 5.98 |
| RNF223 | 3 | 2 | 5.9 |
| TH | 3 | 2 | 5.81 |
| SLC37A3 | 4 | 1 | 5.73 |
| BMP4 | 5 | 0 | 5.61 |
| C2orf50 | 4 | 1 | 5.6 |
| DEGS1 | 4 | 1 | 5.57 |
| MGST2 | 3 | 2 | 5.53 |
| TIFA | 3 | 2 | 5.46 |
| CBX4 | 3 | 2 | 5.36 |
| AP000769.1 | 4 | 1 | 5.31 |
| PIM3 | 4 | 1 | 5.25 |
| PLCD3 | 3 | 2 | 5.25 |
| PDK4 | 3 | 2 | 5.17 |
| CYB561 | 3 | 2 | 5.13 |
| FAM105A | 3 | 2 | 5.05 |
| LEKR1 | 3 | 2 | 4.75 |
| PROSER2 | 3 | 2 | 4.45 |
| STEAP4 | 2 | 3 | 4.36 |
| PKIA | 2 | 3 | 4.22 |
| ANXA9 | 2 | 3 | 4.22 |
| AC091801.1 | 3 | 2 | 4.11 |
| KRT24 | 3 | 2 | 4.1 |
| HAS2 | 4 | 1 | 4.08 |
| ERMP1 | 3 | 2 | 4.07 |
| PAWR | 3 | 2 | 4.03 |
| ANKH | 3 | 2 | 3.88 |
| C17orf82 | 4 | 1 | 3.88 |
| HS1BP3 | 3 | 2 | 3.87 |
| MLYCD | 3 | 2 | 3.86 |
| MYBL2 | 3 | 2 | 3.78 |
| B3GNT3 | 3 | 2 | 3.74 |
| LRRN2 | 4 | 1 | 3.73 |
| KIF3C | 3 | 2 | 3.71 |
| ATOH8 | 3 | 2 | 3.69 |
| TMCC3 | 3 | 2 | 3.67 |
| CRY1 | 1 | 4 | 3.66 |
| SLC39A8 | 2 | 3 | 3.62 |
| MSMB | 2 | 3 | 3.6 |
| OSGIN1 | 3 | 2 | 3.59 |
| B3GNT6 | 2 | 3 | 3.42 |
| CCDC174 | 2 | 3 | 3.38 |
| TNFSF10 | 3 | 2 | 3.33 |
| ATF3 | 3 | 2 | 3.32 |
| TRAK1 | 2 | 3 | 3.21 |
| CPN2 | 3 | 2 | 3.21 |
| ZNF282 | 2 | 3 | 3.2 |
| TMEM64 | 3 | 2 | 3.2 |
| S1PR3 | 3 | 2 | 3.17 |
| BMP5 | 2 | 3 | 3.15 |
| ATP8B2 | 3 | 2 | 3.14 |
| SLC39A10 | 2 | 3 | 3.1 |
| EZR | 2 | 3 | 3.1 |
| RP11-385D13.1 | 2 | 3 | 3.08 |
| SHQ1 | 3 | 2 | 2.98 |
| IL12A | 2 | 3 | 2.92 |
| WISP2 | 3 | 2 | 2.91 |
| INADL | 2 | 3 | 2.9 |
| CLVS1 | 2 | 3 | 2.89 |
| FBXL18 | 2 | 3 | 2.81 |
| ZNF395 | 2 | 3 | 2.78 |
| DDHD1 | 2 | 3 | 2.71 |
| C10orf90 | 2 | 3 | 2.7 |
| MTURN | 2 | 3 | 2.69 |
| AC008271.1 | 2 | 3 | 2.54 |
| CERK | 2 | 3 | 2.44 |
| SPRED2 | 2 | 3 | 2.35 |
| MYO1B | 2 | 3 | 2.34 |
| CRIP2 | 2 | 3 | 2.34 |
| CD55 | 2 | 3 | 2.31 |
| CDHR3 | 2 | 3 | 2.23 |
| ABCA1 | 2 | 3 | 2.23 |
| AZIN1 | 2 | 3 | 2.2 |
| ADRBK1 | 2 | 3 | 2.17 |
| CASP7 | 1 | 4 | 2.13 |
| RRBP1 | 2 | 3 | 2.12 |
| KYNU | 2 | 3 | 2.1 |
| FAM155A | 1 | 4 | 2.09 |
| TFCP2L1 | 2 | 3 | 2.09 |
| SPIN1 | 2 | 3 | 2.08 |
| MESP2 | 1 | 4 | 2.07 |
| SPARCL1 | 2 | 3 | 2.02 |
| CSRNP1 | 2 | 3 | 1.98 |
| KIAA0196 | 2 | 3 | 1.96 |
| LDB3 | 2 | 3 | 1.95 |
| PARP8 | 1 | 4 | 1.94 |
| SUCLG2 | 1 | 4 | 1.94 |
| FAM214A | 1 | 4 | 1.93 |
| PLEKHH2 | 1 | 4 | 1.91 |
| THBS4 | 2 | 3 | 1.91 |
| TMPRSS11A | 1 | 4 | 1.86 |
| GPR64 | 1 | 4 | 1.86 |
| AGR2 | 2 | 3 | 1.82 |
| PERP | 1 | 4 | 1.79 |
| GLI3 | 1 | 4 | 1.78 |
| WIBG | 2 | 3 | 1.77 |
| PKNOX1 | 2 | 3 | 1.74 |
| SLC29A3 | 1 | 4 | 1.73 |
| ATP6V0D2 | 2 | 3 | 1.72 |
| DPY19L1 | 1 | 4 | 1.63 |
| WDR34 | 1 | 4 | 1.63 |
| FAT1 | 1 | 4 | 1.55 |
| ASS1 | 1 | 4 | 1.54 |
| RP11-758M4.1 | 1 | 4 | 1.52 |
| CNDP1 | 1 | 4 | 1.51 |
| AP1B1 | 1 | 4 | 1.48 |
| SNX14 | 1 | 4 | 1.43 |
| KCNJ12 | 1 | 4 | 1.37 |
| CDC42EP1 | 1 | 4 | 1.36 |
| SYNE3 | 1 | 4 | 1.33 |
| PPTC7 | 1 | 4 | 1.31 |
| C9orf91 | 1 | 4 | 1.29 |
| TTC40 | 1 | 4 | 1.25 |
| RUVBL1 | 1 | 4 | 1.24 |
| LAPTM4B | 1 | 4 | 1.23 |
| IGF2BP1 | 1 | 4 | 1.23 |
| IDH2 | 1 | 4 | 1.22 |
| FLVCR2 | 1 | 4 | 1.19 |
| ALDH1L1 | 1 | 4 | 1.18 |
| DKK3 | 1 | 4 | 1.17 |
| PITPNB | 1 | 4 | 1.13 |
| FAM171A1 | 1 | 4 | 1.13 |
| FNBP1 | 1 | 4 | 1.12 |
| TLE4 | 1 | 4 | 1.11 |
| PROM1 | 1 | 4 | 1.08 |
| ASCC1 | 1 | 4 | 1.08 |
| C12orf42 | 1 | 4 | 1.08 |
| COTL1 | 1 | 4 | 1.07 |
| TOP1 | 1 | 4 | 1.03 |
| SNAP25 | 1 | 4 | 1.01 |
| ARHGAP40 | 1 | 4 | 1 |
| DOT1L | 1 | 4 | 0.98 |
| JUP | 1 | 4 | 0.94 |
| RPH3AL | 1 | 4 | 0.93 |
| ARFGAP3 | 1 | 4 | 0.91 |
| NUFIP1 | 1 | 4 | 0.91 |
| MN1 | 1 | 4 | 0.9 |
| TK2 | 1 | 4 | 0.9 |
| RHOD | 1 | 4 | 0.9 |
| PBX4 | 1 | 4 | 0.89 |
| GDF15 | 1 | 4 | 0.88 |
| CACNG1 | 1 | 4 | 0.88 |
| IFLTD1 | 1 | 4 | 0.88 |
| CDK18 | 1 | 4 | 0.88 |
| AOX1 | 1 | 4 | 0.88 |
| SNX8 | 1 | 4 | 0.88 |
| DGKE | 1 | 4 | 0.87 |
| GCNT1 | 1 | 4 | 0.87 |
| UBE2E2 | 1 | 4 | 0.87 |
| MLXIP | 1 | 4 | 0.86 |
| TMOD3 | 1 | 4 | 0.85 |
| PDIA5 | 1 | 4 | 0.85 |
| PLCG1 | 1 | 4 | 0.85 |
| PTGIS | 1 | 4 | 0.84 |
| AGPAT3 | 1 | 4 | 0.84 |
| PPP2R5E | 1 | 4 | 0.82 |
| SHROOM2 | 1 | 4 | 0.82 |
| AFAP1 | 1 | 4 | 0.82 |
| RAB15 | 1 | 4 | 0.8 |
| GAS7 | 1 | 4 | 0.8 |
| CD101 | 1 | 4 | 0.79 |
| TCF7L1 | 1 | 4 | 0.79 |
| CDS2 | 1 | 3 | 21.36 |
| OSMR | 3 | 1 | 9.67 |
| TACSTD2 | 3 | 1 | 8.9 |
| SMYD2 | 3 | 1 | 8.63 |
| LBH | 3 | 1 | 8.12 |
| TPM1 | 4 | 0 | 7.19 |
| PMAIP1 | 4 | 0 | 7.16 |
| HEY1 | 3 | 1 | 7 |
| PABPC4L | 4 | 0 | 6.93 |
| FOXC1 | 3 | 1 | 6.81 |
| HILPDA | 4 | 0 | 6.52 |
| EFCAB10 | 2 | 2 | 6.48 |
| C7orf65 | 4 | 0 | 6.32 |
| FST | 2 | 2 | 6.09 |
| COMTD1 | 2 | 2 | 5.83 |
| COX6C | 2 | 2 | 5.81 |
| USP25 | 3 | 1 | 5.77 |
| SLC2A8 | 3 | 1 | 5.74 |
| CCDC74A | 4 | 0 | 5.6 |
| PSD4 | 3 | 1 | 5.57 |
| AC021218.2 | 4 | 0 | 5.42 |
| PTMA | 3 | 1 | 5.14 |
| IKZF2 | 3 | 1 | 5.04 |
| MPP7 | 2 | 2 | 5.03 |
| VCAN | 2 | 2 | 5.01 |
| FHDC1 | 3 | 1 | 4.97 |
| SPATA12 | 3 | 1 | 4.88 |
| RBM15 | 3 | 1 | 4.87 |
| RPLP1 | 4 | 0 | 4.81 |
| AKR1C2 | 3 | 1 | 4.74 |
| AL356356.1 | 3 | 1 | 4.71 |
| PRDM7 | 3 | 1 | 4.61 |
| THBS2 | 3 | 1 | 4.51 |
| CWH43 | 4 | 0 | 4.46 |
| DKFZP667F0711 | 4 | 0 | 4.41 |
| TCN1 | 2 | 2 | 4.36 |
| WBP4 | 2 | 2 | 4.32 |
| GPR65 | 3 | 1 | 4.2 |
| LSMEM1 | 3 | 1 | 4.13 |
| ESRP2 | 3 | 1 | 3.99 |
| YTHDF1 | 2 | 2 | 3.97 |
| VAC14 | 3 | 1 | 3.95 |
| P2RX4 | 2 | 2 | 3.86 |
| DECR1 | 2 | 2 | 3.84 |
| SLC25A1 | 4 | 0 | 3.77 |
| NHLRC1 | 3 | 1 | 3.75 |
| EMILIN3 | 3 | 1 | 3.7 |
| PPM1B | 3 | 1 | 3.63 |
| TRIM17 | 3 | 1 | 3.63 |
| CHD2 | 3 | 1 | 3.6 |
| RAD23B | 3 | 1 | 3.57 |
| PGRMC2 | 4 | 0 | 3.54 |
| PARL | 2 | 2 | 3.5 |
| PPP1R3B | 3 | 1 | 3.49 |
| SLC13A1 | 2 | 2 | 3.49 |
| XBP1 | 3 | 1 | 3.39 |
| ZSCAN2 | 1 | 3 | 3.38 |
| INHA | 3 | 1 | 3.38 |
| SLC37A1 | 2 | 2 | 3.37 |
| TRIB1 | 2 | 2 | 3.36 |
| FECH | 3 | 1 | 3.31 |
| OCIAD2 | 3 | 1 | 3.26 |
| EPC1 | 2 | 2 | 3.19 |
| WSB1 | 3 | 1 | 3.17 |
| C17orf103 | 2 | 2 | 3.08 |
| MAP3K7 | 3 | 1 | 3.03 |
| SMPDL3A | 2 | 2 | 3.02 |
| ARHGEF16 | 2 | 2 | 3.01 |
| KIF5B | 3 | 1 | 2.96 |
| TIPARP | 2 | 2 | 2.85 |
| FAM46C | 2 | 2 | 2.85 |
| TMEM43 | 2 | 2 | 2.84 |
| NBPF4 | 3 | 1 | 2.81 |
| STK38L | 3 | 1 | 2.79 |
| TARS | 2 | 2 | 2.79 |
| EPHA2 | 2 | 2 | 2.69 |
| PCYT1A | 3 | 1 | 2.68 |
| ITGB6 | 3 | 1 | 2.65 |
| DST | 3 | 1 | 2.64 |
| TMEM71 | 2 | 2 | 2.63 |
| SLC46A2 | 2 | 2 | 2.53 |
| MANBA | 2 | 2 | 2.5 |
| KRT37 | 2 | 2 | 2.39 |
| WFS1 | 2 | 2 | 2.36 |
| TUBGCP3 | 2 | 2 | 2.34 |
| HNMT | 2 | 2 | 2.32 |
| SP3 | 2 | 2 | 2.32 |
| PLIN3 | 2 | 2 | 2.32 |
| TPD52 | 2 | 2 | 2.31 |
| FADD | 2 | 2 | 2.3 |
| KEAP1 | 1 | 3 | 2.28 |
| SEPT10 | 2 | 2 | 2.25 |
| GMFB | 2 | 2 | 2.22 |
| CTGF | 1 | 3 | 2.19 |
| LIPH | 1 | 3 | 2.17 |
| PRMT6 | 1 | 3 | 2.16 |
| RNF130 | 2 | 2 | 2.15 |
| GPRC5C | 2 | 2 | 2.14 |
| SERINC5 | 1 | 3 | 2.08 |
| AXDND1 | 2 | 2 | 2.06 |
| GPRIN2 | 2 | 2 | 2.06 |
| ENC1 | 1 | 3 | 2.03 |
| DISC1 | 2 | 2 | 2.01 |
| SLCO4A1 | 1 | 3 | 2.01 |
| C9orf64 | 2 | 2 | 1.99 |
| ENPP2 | 2 | 2 | 1.98 |
| ZBTB10 | 2 | 2 | 1.98 |
| SCHIP1 | 1 | 3 | 1.98 |
| RTN4IP1 | 2 | 2 | 1.93 |
| PSMA3 | 1 | 3 | 1.93 |
| HECW2 | 1 | 3 | 1.88 |
| AP4E1 | 2 | 2 | 1.86 |
| LONRF2 | 1 | 3 | 1.85 |
| RPS6KA2 | 2 | 2 | 1.82 |
| ARSJ | 1 | 3 | 1.82 |
| FAM49A | 2 | 2 | 1.81 |
| CECR6 | 1 | 3 | 1.79 |
| SLC19A2 | 2 | 2 | 1.78 |
| SLC6A17 | 2 | 2 | 1.77 |
| FAM213A | 2 | 2 | 1.76 |
| GPR160 | 2 | 2 | 1.74 |
| IQSEC2 | 1 | 3 | 1.73 |
| TNFRSF13B | 2 | 2 | 1.73 |
| TOR1AIP2 | 1 | 3 | 1.73 |
| C8orf46 | 2 | 2 | 1.73 |
| ZNF516 | 1 | 3 | 1.7 |
| CGN | 1 | 3 | 1.65 |
| KBTBD11 | 1 | 3 | 1.58 |
| GRB14 | 1 | 3 | 1.56 |
| C12orf79 | 1 | 3 | 1.55 |
| LMX1A | 1 | 3 | 1.54 |
| DNAJC12 | 1 | 3 | 1.53 |
| PDE8B | 1 | 3 | 1.48 |
| WISP1 | 1 | 3 | 1.47 |
| ADIPOR2 | 1 | 3 | 1.46 |
| ALS2CL | 1 | 3 | 1.42 |
| CUBN | 1 | 3 | 1.39 |
| HSDL2 | 1 | 3 | 1.38 |
| TECR | 1 | 3 | 1.38 |
| CSK | 1 | 3 | 1.37 |
| KCNA2 | 1 | 3 | 1.36 |
| PCYT2 | 1 | 3 | 1.34 |
| LOXL4 | 1 | 3 | 1.34 |
| ACOX3 | 1 | 3 | 1.32 |
| CCDC125 | 1 | 3 | 1.31 |
| EDN3 | 1 | 3 | 1.31 |
| TSPAN1 | 1 | 3 | 1.3 |
| ARID3B | 1 | 3 | 1.26 |
| SAMD8 | 1 | 3 | 1.26 |
| KPNA7 | 1 | 3 | 1.25 |
| C5orf28 | 1 | 3 | 1.25 |
| MAP2K1 | 1 | 3 | 1.23 |
| KRT86 | 1 | 3 | 1.22 |
| OSBPL9 | 1 | 3 | 1.21 |
| KIAA0040 | 1 | 3 | 1.2 |
| PDZK1 | 1 | 3 | 1.19 |
| PWWP2B | 1 | 3 | 1.18 |
| SYTL4 | 1 | 3 | 1.18 |
| AL359878.1 | 1 | 3 | 1.18 |
| SDC2 | 1 | 3 | 1.12 |
| CPE | 1 | 3 | 1.1 |
| SPDEF | 1 | 3 | 1.1 |
| TRAM2 | 1 | 3 | 1.1 |
| CSPG4 | 1 | 3 | 1.09 |
| MSI1 | 1 | 3 | 1.08 |
| AGPS | 1 | 3 | 1.07 |
| NIPAL2 | 1 | 3 | 1.07 |
| SNX24 | 1 | 3 | 1.06 |
| ABLIM3 | 1 | 3 | 1.05 |
| KHNYN | 1 | 3 | 1.03 |
| COLQ | 1 | 3 | 1.03 |
| CDA | 1 | 3 | 1.02 |
| MDM2 | 1 | 3 | 1.02 |
| FAIM3 | 1 | 3 | 1.01 |
| CTD-3088G3.8 | 1 | 3 | 1.01 |
| RALGAPA2 | 1 | 3 | 1.01 |
| AMOTL1 | 1 | 3 | 1.01 |
| ARHGAP35 | 1 | 3 | 0.98 |
| LIMS1 | 1 | 3 | 0.98 |
| PLXND1 | 1 | 3 | 0.97 |
| RIN2 | 1 | 3 | 0.96 |
| CYFIP1 | 1 | 3 | 0.96 |
| RALB | 1 | 3 | 0.96 |
| FREM1 | 1 | 3 | 0.96 |
| GPR132 | 1 | 3 | 0.96 |
| PGAP2 | 1 | 3 | 0.95 |
| EMILIN2 | 1 | 3 | 0.94 |
| DNAH14 | 1 | 3 | 0.92 |
| HSPG2 | 1 | 3 | 0.92 |
| BMPR2 | 1 | 3 | 0.91 |
| WDR72 | 1 | 3 | 0.91 |
| NTRK3 | 1 | 3 | 0.9 |
| NPR3 | 1 | 3 | 0.89 |
| DTL | 1 | 3 | 0.89 |
| ASF1B | 1 | 3 | 0.88 |
| SCARA3 | 1 | 3 | 0.88 |
| PCTP | 1 | 3 | 0.86 |
| PEMT | 1 | 3 | 0.86 |
| PAK2 | 1 | 3 | 0.86 |
| KIT | 1 | 3 | 0.85 |
| TFF1 | 1 | 3 | 0.84 |
| SLC47A1 | 1 | 3 | 0.83 |
| BMP1 | 1 | 3 | 0.83 |
| PLAGL1 | 1 | 3 | 0.83 |
| KIAA0513 | 1 | 3 | 0.82 |
| SLC13A4 | 1 | 3 | 0.81 |
| FSIP2 | 1 | 3 | 0.8 |
| UPK3A | 3 | 0 | 7 |
| BCL10 | 3 | 0 | 5.76 |
| PPIF | 3 | 0 | 5.51 |
| ARSD | 2 | 1 | 4.83 |
| POP1 | 3 | 0 | 4.75 |
| HCCS | 3 | 0 | 4.68 |
| JADE2 | 2 | 1 | 4.49 |
| B3GNT2 | 2 | 1 | 4.41 |
| MBD2 | 2 | 1 | 4.41 |
| MUC20 | 3 | 0 | 4.36 |
| SLC2A1 | 2 | 1 | 4.24 |
| AC003101.1 | 2 | 1 | 4.01 |
| SLC38A2 | 2 | 1 | 3.99 |
| PDLIM5 | 3 | 0 | 3.95 |
| PLET1 | 2 | 1 | 3.92 |
| OR13H1 | 3 | 0 | 3.91 |
| TSPYL5 | 3 | 0 | 3.84 |
| PTP4A1 | 2 | 1 | 3.83 |
| LAPTM4A | 2 | 1 | 3.78 |
| WNT16 | 2 | 1 | 3.67 |
| ONECUT1 | 3 | 0 | 3.64 |
| PIM1 | 2 | 1 | 3.63 |
| C8orf4 | 3 | 0 | 3.63 |
| ACE | 2 | 1 | 3.46 |
| POU5F1B | 3 | 0 | 3.44 |
| FAM129A | 2 | 1 | 3.43 |
| ANKRD26 | 2 | 1 | 3.43 |
| HSBP1 | 3 | 0 | 3.41 |
| MBP | 3 | 0 | 3.38 |
| SPATA31D1 | 3 | 0 | 3.31 |
| GABRP | 3 | 0 | 3.29 |
| KLHL25 | 3 | 0 | 3.25 |
| MPST | 2 | 1 | 3.25 |
| PHLDA1 | 3 | 0 | 3.25 |
| RASGRP1 | 2 | 1 | 3.21 |
| CLN8 | 1 | 2 | 3.2 |
| KLHL4 | 2 | 1 | 3.18 |
| RP11-187E13.1 | 3 | 0 | 3.18 |
| LDHD | 2 | 1 | 3.18 |
| DHRS2 | 2 | 1 | 3.17 |
| HIVEP2 | 2 | 1 | 3.13 |
| NCF2 | 2 | 1 | 3.12 |
| FOXS1 | 2 | 1 | 3.12 |
| ZFP36L1 | 3 | 0 | 3.09 |
| ATP6AP2 | 3 | 0 | 3.06 |
| GBE1 | 2 | 1 | 3.03 |
| SCCPDH | 2 | 1 | 3.03 |
| CRYAA | 3 | 0 | 2.96 |
| C2CD4A | 2 | 1 | 2.94 |
| ACER2 | 1 | 2 | 2.92 |
| MOB2 | 1 | 2 | 2.89 |
| KLF7 | 2 | 1 | 2.78 |
| SUSD2 | 3 | 0 | 2.74 |
| CALML5 | 3 | 0 | 2.73 |
| SHH | 3 | 0 | 2.72 |
| LY6D | 2 | 1 | 2.7 |
| ENTPD3 | 1 | 2 | 2.68 |
| PTGS1 | 1 | 2 | 2.64 |
| KMT2E | 2 | 1 | 2.63 |
| HDLBP | 2 | 1 | 2.61 |
| TMEM105 | 2 | 1 | 2.6 |
| TGIF1 | 2 | 1 | 2.59 |
| RP11-650K20.3 | 2 | 1 | 2.55 |
| DYNLRB2 | 2 | 1 | 2.53 |
| EFCAB2 | 1 | 2 | 2.53 |
| MOCS1 | 1 | 2 | 2.5 |
| BCL3 | 2 | 1 | 2.49 |
| ETV3 | 2 | 1 | 2.49 |
| OGFRL1 | 2 | 1 | 2.49 |
| CD22 | 2 | 1 | 2.48 |
| OSGIN2 | 2 | 1 | 2.46 |
| CCRN4L | 2 | 1 | 2.44 |
| SLC35F6 | 1 | 2 | 2.44 |
| DHX15 | 2 | 1 | 2.37 |
| KCTD21 | 2 | 1 | 2.36 |
| ARHGEF39 | 2 | 1 | 2.36 |
| PLEKHF2 | 2 | 1 | 2.26 |
| NDUFS6 | 2 | 1 | 2.25 |
| TECTB | 2 | 1 | 2.23 |
| MRPS10 | 2 | 1 | 2.23 |
| NBEA | 1 | 2 | 2.23 |
| GHRHR | 2 | 1 | 2.21 |
| ACOT6 | 2 | 1 | 2.18 |
| PKP2 | 2 | 1 | 2.17 |
| LRRC58 | 2 | 1 | 2.16 |
| GPRC5A | 2 | 1 | 2.15 |
| VIT | 1 | 2 | 2.14 |
| FAM83H | 1 | 2 | 2.14 |
| LPIN3 | 1 | 2 | 2.11 |
| SLC46A3 | 1 | 2 | 2.09 |
| GATA5 | 2 | 1 | 2.08 |
| C4orf26 | 1 | 2 | 2.06 |
| VRK2 | 2 | 1 | 2.04 |
| MAMLD1 | 2 | 1 | 2.04 |
| SIX1 | 2 | 1 | 1.99 |
| FOXN4 | 2 | 1 | 1.98 |
| RAB36 | 1 | 2 | 1.96 |
| SGCG | 2 | 1 | 1.95 |
| KPNA3 | 2 | 1 | 1.91 |
| TRMT61A | 1 | 2 | 1.9 |
| PSMA2 | 2 | 1 | 1.86 |
| ABHD16B | 2 | 1 | 1.84 |
| RABGEF1 | 1 | 2 | 1.83 |
| UBE2H | 1 | 2 | 1.83 |
| HLA-DRB1 | 2 | 1 | 1.79 |
| SUSD3 | 2 | 1 | 1.79 |
| NELFA | 1 | 2 | 1.75 |
| HERC3 | 1 | 2 | 1.73 |
| ATP5G3 | 2 | 1 | 1.73 |
| NBN | 1 | 2 | 1.71 |
| CEP135 | 1 | 2 | 1.71 |
| SLC19A3 | 1 | 2 | 1.7 |
| TTC39A | 2 | 1 | 1.68 |
| RGS8 | 1 | 2 | 1.68 |
| MRPS18A | 1 | 2 | 1.62 |
| PLAC8 | 1 | 2 | 1.6 |
| HR | 1 | 2 | 1.59 |
| CBR3 | 2 | 1 | 1.59 |
| HID1 | 1 | 2 | 1.53 |
| MTMR1 | 1 | 2 | 1.53 |
| PYROXD2 | 1 | 2 | 1.51 |
| DTWD2 | 1 | 2 | 1.51 |
| LIPA | 1 | 2 | 1.5 |
| CXorf58 | 1 | 2 | 1.48 |
| MLLT4 | 1 | 2 | 1.44 |
| ZG16 | 1 | 2 | 1.42 |
| EVA1A | 1 | 2 | 1.4 |
| ILVBL | 1 | 2 | 1.38 |
| AKAP7 | 1 | 2 | 1.38 |
| MYL12B | 1 | 2 | 1.38 |
| CAMK1G | 1 | 2 | 1.38 |
| METTL20 | 1 | 2 | 1.38 |
| COBL | 1 | 2 | 1.38 |
| GINS2 | 1 | 2 | 1.35 |
| CHGA | 1 | 2 | 1.35 |
| PLXNA1 | 1 | 2 | 1.35 |
| DIRC1 | 1 | 2 | 1.32 |
| IMPDH1 | 1 | 2 | 1.31 |
| CDKL1 | 1 | 2 | 1.28 |
| WNT7B | 1 | 2 | 1.28 |
| NQO1 | 1 | 2 | 1.28 |
| NR1D2 | 1 | 2 | 1.28 |
| SPCS3 | 1 | 2 | 1.27 |
| NMNAT1 | 1 | 2 | 1.26 |
| LHFPL2 | 1 | 2 | 1.25 |
| SCG5 | 1 | 2 | 1.25 |
| MAP3K4 | 1 | 2 | 1.25 |
| C1orf170 | 1 | 2 | 1.23 |
| S1PR2 | 1 | 2 | 1.2 |
| PSPC1 | 1 | 2 | 1.19 |
| PRSS22 | 1 | 2 | 1.18 |
| GULP1 | 1 | 2 | 1.18 |
| RNF213 | 1 | 2 | 1.17 |
| SYCP2 | 1 | 2 | 1.17 |
| ELF1 | 1 | 2 | 1.17 |
| DEF6 | 1 | 2 | 1.16 |
| ADPRHL1 | 1 | 2 | 1.16 |
| CYB5A | 1 | 2 | 1.15 |
| TMEM57 | 1 | 2 | 1.15 |
| NNT | 1 | 2 | 1.14 |
| DIXDC1 | 1 | 2 | 1.14 |
| MPP5 | 1 | 2 | 1.11 |
| FAM25A | 1 | 2 | 1.1 |
| ART4 | 1 | 2 | 1.1 |
| ZBTB37 | 1 | 2 | 1.09 |
| MIPEP | 1 | 2 | 1.09 |
| ZNF107 | 1 | 2 | 1.08 |
| ATP2B1 | 1 | 2 | 1.08 |
| IBTK | 1 | 2 | 1.08 |
| PET112 | 1 | 2 | 1.08 |
| TEF | 1 | 2 | 1.08 |
| KIF13B | 1 | 2 | 1.08 |
| NCOA4 | 1 | 2 | 1.07 |
| CEBPB | 1 | 2 | 1.06 |
| STRN4 | 1 | 2 | 1.06 |
| GDF6 | 1 | 2 | 1.05 |
| GAPVD1 | 1 | 2 | 1.05 |
| KRT80 | 1 | 2 | 1.05 |
| LAMA4 | 1 | 2 | 1.04 |
| HAO2 | 1 | 2 | 1.03 |
| ABCD2 | 1 | 2 | 1.02 |
| CABLES2 | 1 | 2 | 1.01 |
| ST6GALNAC1 | 1 | 2 | 1.01 |
| PRKAA1 | 1 | 2 | 1.01 |
| NKD2 | 1 | 2 | 1 |
| VSIG10 | 1 | 2 | 1 |
| ZFP64 | 1 | 2 | 1 |
| AQP3 | 1 | 2 | 1 |
| C5orf64 | 1 | 2 | 0.99 |
| MCU | 1 | 2 | 0.99 |
| SMTNL2 | 1 | 2 | 0.99 |
| ADAMTS16 | 1 | 2 | 0.99 |
| FLT1 | 1 | 2 | 0.98 |
| TNS1 | 1 | 2 | 0.98 |
| LYZL2 | 1 | 2 | 0.98 |
| NGF | 1 | 2 | 0.96 |
| ITGB2 | 1 | 2 | 0.96 |
| GCLC | 1 | 2 | 0.95 |
| EEA1 | 1 | 2 | 0.95 |
| GM2A | 1 | 2 | 0.94 |
| MMP16 | 1 | 2 | 0.94 |
| ZBTB7C | 1 | 2 | 0.94 |
| SLC45A1 | 1 | 2 | 0.93 |
| BBIP1 | 1 | 2 | 0.92 |
| LDLR | 1 | 2 | 0.92 |
| MAP4K3 | 1 | 2 | 0.92 |
| TRIP13 | 1 | 2 | 0.91 |
| KCNC1 | 1 | 2 | 0.91 |
| TACR1 | 1 | 2 | 0.9 |
| SYT15 | 1 | 2 | 0.89 |
| SH3BGRL2 | 1 | 2 | 0.89 |
| RDH10 | 1 | 2 | 0.89 |
| ZNF195 | 1 | 2 | 0.88 |
| GAA | 1 | 2 | 0.87 |
| CHI3L1 | 1 | 2 | 0.87 |
| TMED2 | 1 | 2 | 0.87 |
| CIPC | 1 | 2 | 0.86 |
| SMIM20 | 1 | 2 | 0.86 |
| BCL9 | 1 | 2 | 0.85 |
| AIFM2 | 1 | 2 | 0.85 |
| XRCC2 | 1 | 2 | 0.84 |
| C12orf75 | 1 | 2 | 0.84 |
| PCMTD1 | 1 | 2 | 0.84 |
| FOXP4 | 1 | 2 | 0.84 |
| ZFYVE28 | 1 | 2 | 0.83 |
| PTP4A3 | 1 | 2 | 0.83 |
| PAX9 | 1 | 2 | 0.83 |
| HIF1A | 1 | 2 | 0.83 |
| RHCG | 1 | 2 | 0.83 |
| XPNPEP1 | 1 | 2 | 0.82 |
| DNAH1 | 1 | 2 | 0.82 |
| OCLN | 1 | 2 | 0.82 |
| FAM19A2 | 1 | 2 | 0.82 |
| ITGA2 | 1 | 2 | 0.82 |
| ZBED4 | 1 | 2 | 0.81 |
| JPH3 | 1 | 2 | 0.81 |
| HERC6 | 1 | 2 | 0.8 |
| DEPTOR | 1 | 2 | 0.8 |
| TESC | 1 | 2 | 0.8 |
| ZBTB5 | 1 | 2 | 0.79 |
| MAL2 | 1 | 2 | 0.79 |
| AC022431.2 | 1 | 2 | 0.79 |
| TBC1D21 | 1 | 2 | 0.79 |
| USH2A | 1 | 2 | 0.79 |
| DKK1 | 2 | 0 | 6.21 |
| FAM110A | 2 | 0 | 5.5 |
| FAM109A | 2 | 0 | 4.75 |
| FRG2C | 2 | 0 | 4.66 |
| SOX3 | 2 | 0 | 4.41 |
| FEZF2 | 2 | 0 | 4.32 |
| SYPL1 | 2 | 0 | 3.97 |
| ID1 | 2 | 0 | 3.72 |
| ID4 | 2 | 0 | 3.7 |
| C1orf143 | 2 | 0 | 3.63 |
| SLC31A2 | 2 | 0 | 3.5 |
| C9orf69 | 2 | 0 | 3.43 |
| CLDN25 | 2 | 0 | 3.41 |
| MICB | 1 | 1 | 3.28 |
| GUSB | 2 | 0 | 3.21 |
| SLITRK6 | 2 | 0 | 3.15 |
| CBWD5 | 1 | 1 | 3.12 |
| RAP2B | 2 | 0 | 3.11 |
| RGS16 | 1 | 1 | 3.07 |
| TNS3 | 2 | 0 | 3.06 |
| STXBP5 | 2 | 0 | 3.03 |
| CTSL | 2 | 0 | 3.03 |
| SLC4A7 | 2 | 0 | 3.02 |
| PANK3 | 2 | 0 | 3.01 |
| KCNF1 | 2 | 0 | 3.01 |
| FAM49B | 2 | 0 | 2.99 |
| PRDX6 | 2 | 0 | 2.98 |
| SIM1 | 2 | 0 | 2.96 |
| PRDM9 | 2 | 0 | 2.95 |
| CALM1 | 2 | 0 | 2.86 |
| RASL11B | 2 | 0 | 2.74 |
| SEMA5A | 2 | 0 | 2.73 |
| PAPD7 | 1 | 1 | 2.72 |
| TUBGCP2 | 1 | 1 | 2.72 |
| BHLHE40 | 2 | 0 | 2.71 |
| GLDC | 1 | 1 | 2.71 |
| KIAA0087 | 2 | 0 | 2.68 |
| ARRDC3 | 2 | 0 | 2.66 |
| AL354993.1 | 2 | 0 | 2.61 |
| GJD4 | 2 | 0 | 2.58 |
| GLRA3 | 2 | 0 | 2.55 |
| HEY2 | 1 | 1 | 2.54 |
| TLR5 | 2 | 0 | 2.53 |
| PCDH7 | 1 | 1 | 2.52 |
| NFIL3 | 1 | 1 | 2.52 |
| CCDC182 | 2 | 0 | 2.51 |
| PDE5A | 2 | 0 | 2.49 |
| IRS2 | 2 | 0 | 2.49 |
| CD180 | 2 | 0 | 2.48 |
| NAT8 | 2 | 0 | 2.48 |
| EFEMP1 | 2 | 0 | 2.39 |
| PDGFA | 2 | 0 | 2.37 |
| CTB-167G5.5 | 2 | 0 | 2.32 |
| ARL15 | 1 | 1 | 2.32 |
| KPNB1 | 1 | 1 | 2.32 |
| RAPH1 | 2 | 0 | 2.32 |
| ANKRD30BL | 2 | 0 | 2.25 |
| PGD | 1 | 1 | 2.24 |
| KIAA1191 | 1 | 1 | 2.23 |
| RHOBTB3 | 2 | 0 | 2.22 |
| C5orf38 | 2 | 0 | 2.21 |
| COL22A1 | 2 | 0 | 2.15 |
| CAV3 | 2 | 0 | 2.13 |
| JUN | 2 | 0 | 2.11 |
| ABCB4 | 2 | 0 | 2.1 |
| XIAP | 2 | 0 | 2.09 |
| ENOPH1 | 1 | 1 | 2.09 |
| COL28A1 | 2 | 0 | 2.09 |
| CRB2 | 2 | 0 | 2.09 |
| NPTX2 | 1 | 1 | 2.08 |
| FAM20B | 2 | 0 | 2.05 |
| RHOV | 1 | 1 | 2.05 |
| ARHGAP12 | 1 | 1 | 2.05 |
| NFE2L2 | 2 | 0 | 2.04 |
| ASTL | 1 | 1 | 2.03 |
| MPC1 | 1 | 1 | 2.03 |
| ZIC2 | 1 | 1 | 2 |
| CD276 | 1 | 1 | 1.98 |
| ZNF318 | 1 | 1 | 1.97 |
| SLC35B4 | 2 | 0 | 1.96 |
| EDN2 | 2 | 0 | 1.96 |
| BTG2 | 2 | 0 | 1.95 |
| SPRY2 | 1 | 1 | 1.94 |
| ADAM30 | 2 | 0 | 1.91 |
| SCAP | 1 | 1 | 1.91 |
| DCSTAMP | 2 | 0 | 1.91 |
| HLA-F | 1 | 1 | 1.89 |
| RPL39L | 1 | 1 | 1.87 |
| CAV2 | 2 | 0 | 1.84 |
| AC124890.1 | 1 | 1 | 1.84 |
| GNLY | 1 | 1 | 1.83 |
| ATG5 | 1 | 1 | 1.83 |
| EIF2S3 | 1 | 1 | 1.81 |
| OR8S1 | 1 | 1 | 1.81 |
| TRHR | 1 | 1 | 1.8 |
| ASPH | 2 | 0 | 1.75 |
| SCP2D1 | 2 | 0 | 1.73 |
| RGS1 | 1 | 1 | 1.73 |
| PPAPDC3 | 1 | 1 | 1.72 |
| SNAI2 | 2 | 0 | 1.72 |
| DUSP1 | 2 | 0 | 1.71 |
| PHF13 | 1 | 1 | 1.71 |
| FKBP4 | 1 | 1 | 1.7 |
| JAG1 | 1 | 1 | 1.68 |
| DLL1 | 2 | 0 | 1.68 |
| SLC7A13 | 2 | 0 | 1.68 |
| HLA-DRB5 | 2 | 0 | 1.68 |
| KAAG1 | 2 | 0 | 1.68 |
| MBL2 | 2 | 0 | 1.66 |
| ZBTB43 | 1 | 1 | 1.66 |
| JUND | 1 | 1 | 1.66 |
| COG1 | 1 | 1 | 1.66 |
| ENDOD1 | 1 | 1 | 1.65 |
| AC016251.1 | 2 | 0 | 1.65 |
| NCCRP1 | 1 | 1 | 1.63 |
| GALNT3 | 2 | 0 | 1.63 |
| PGLYRP2 | 1 | 1 | 1.63 |
| RP11-187E13.2 | 1 | 1 | 1.62 |
| DNM1L | 1 | 1 | 1.61 |
| AGPAT6 | 1 | 1 | 1.6 |
| APPL2 | 1 | 1 | 1.57 |
| CHCHD5 | 1 | 1 | 1.56 |
| SFRP1 | 1 | 1 | 1.54 |
| EMP1 | 1 | 1 | 1.51 |
| C15orf38 | 1 | 1 | 1.51 |
| C4orf36 | 1 | 1 | 1.5 |
| PTHLH | 1 | 1 | 1.5 |
| GRIK3 | 1 | 1 | 1.49 |
| AL158147.2 | 1 | 1 | 1.48 |
| HOOK1 | 1 | 1 | 1.48 |
| IFNGR1 | 1 | 1 | 1.48 |
| RARS2 | 1 | 1 | 1.47 |
| MRPL15 | 1 | 1 | 1.47 |
| AP3B2 | 1 | 1 | 1.46 |
| SFTPD | 1 | 1 | 1.45 |
| CCL17 | 1 | 1 | 1.45 |
| SIRT5 | 1 | 1 | 1.44 |
| FAM217A | 1 | 1 | 1.44 |
| FKBP9 | 1 | 1 | 1.44 |
| RHOBTB1 | 1 | 1 | 1.39 |
| CDK17 | 1 | 1 | 1.39 |
| KCNJ3 | 1 | 1 | 1.38 |
| SEMA3E | 1 | 1 | 1.36 |
| PROX1 | 1 | 1 | 1.36 |
| OCIAD1 | 1 | 1 | 1.35 |
| MSRB2 | 1 | 1 | 1.35 |
| DAPL1 | 1 | 1 | 1.34 |
| CDKN1A | 1 | 1 | 1.34 |
| RALGDS | 1 | 1 | 1.34 |
| MAP1LC3B2 | 1 | 1 | 1.33 |
| LMTK2 | 1 | 1 | 1.31 |
| PTPRH | 1 | 1 | 1.31 |
| FAM160B1 | 1 | 1 | 1.31 |
| SLC25A5 | 1 | 1 | 1.3 |
| PTPLA | 1 | 1 | 1.3 |
| KANSL3 | 1 | 1 | 1.3 |
| TSHR | 1 | 1 | 1.3 |
| TRMT12 | 1 | 1 | 1.3 |
| NXT2 | 1 | 1 | 1.3 |
| CREB1 | 1 | 1 | 1.29 |
| RBM11 | 1 | 1 | 1.29 |
| ATP8B4 | 1 | 1 | 1.28 |
| LRRC32 | 1 | 1 | 1.28 |
| EVX2 | 1 | 1 | 1.28 |
| NFE2L1 | 1 | 1 | 1.27 |
| INSIG1 | 1 | 1 | 1.27 |
| SLC45A4 | 1 | 1 | 1.27 |
| CCSER2 | 1 | 1 | 1.27 |
| ZNF706 | 1 | 1 | 1.25 |
| TSPAN10 | 1 | 1 | 1.25 |
| BTC | 1 | 1 | 1.25 |
| GUK1 | 1 | 1 | 1.24 |
| MCEE | 1 | 1 | 1.24 |
| ANXA2R | 1 | 1 | 1.22 |
| LRIG3 | 1 | 1 | 1.22 |
| ABCD3 | 1 | 1 | 1.22 |
| SSBP2 | 1 | 1 | 1.21 |
| LEO1 | 1 | 1 | 1.21 |
| IRF4 | 1 | 1 | 1.2 |
| ITGA4 | 1 | 1 | 1.2 |
| PRR15 | 1 | 1 | 1.2 |
| APOA1 | 1 | 1 | 1.19 |
| CLDN9 | 1 | 1 | 1.18 |
| SPTSSA | 1 | 1 | 1.18 |
| ANXA10 | 1 | 1 | 1.17 |
| PP13004 | 1 | 1 | 1.16 |
| ALAD | 1 | 1 | 1.16 |
| ATP1A2 | 1 | 1 | 1.16 |
| PDE1A | 1 | 1 | 1.16 |
| C5orf42 | 1 | 1 | 1.16 |
| GLA | 1 | 1 | 1.16 |
| PIP5KL1 | 1 | 1 | 1.15 |
| SLC1A5 | 1 | 1 | 1.14 |
| MACC1 | 1 | 1 | 1.13 |
| PLEKHF1 | 1 | 1 | 1.13 |
| TUBA3E | 1 | 1 | 1.13 |
| NEIL2 | 1 | 1 | 1.12 |
| C17orf98 | 1 | 1 | 1.11 |
| BOD1 | 1 | 1 | 1.11 |
| UGT2A3 | 1 | 1 | 1.11 |
| EFNB2 | 1 | 1 | 1.1 |
| WDR1 | 1 | 1 | 1.1 |
| CNDP2 | 1 | 1 | 1.1 |
| CD83 | 1 | 1 | 1.1 |
| ZNF185 | 1 | 1 | 1.09 |
| FKBP1A | 1 | 1 | 1.09 |
| ERI1 | 1 | 1 | 1.08 |
| FOXO6 | 1 | 1 | 1.08 |
| FAM60A | 1 | 1 | 1.08 |
| NDUFV2 | 1 | 1 | 1.08 |
| VAPA | 1 | 1 | 1.07 |
| HMGN4 | 1 | 1 | 1.07 |
| SOX9 | 1 | 1 | 1.07 |
| RBM12B-AS1 | 1 | 1 | 1.07 |
| CXXC11 | 1 | 1 | 1.07 |
| IER5L | 1 | 1 | 1.07 |
| ZFPM2 | 1 | 1 | 1.05 |
| PPM1J | 1 | 1 | 1.05 |
| PLK2 | 1 | 1 | 1.04 |
| SERPINE1 | 1 | 1 | 1.04 |
| GCM1 | 1 | 1 | 1.03 |
| EPHB6 | 1 | 1 | 1.02 |
| TBC1D10C | 1 | 1 | 1.02 |
| ROCK2 | 1 | 1 | 1.02 |
| CST5 | 1 | 1 | 1.01 |
| AC093802.1 | 1 | 1 | 1.01 |
| WDR60 | 1 | 1 | 1 |
| MIEF2 | 1 | 1 | 1 |
| NSG2 | 1 | 1 | 0.99 |
| MAP3K1 | 1 | 1 | 0.99 |
| GPR20 | 1 | 1 | 0.98 |
| AWAT1 | 1 | 1 | 0.98 |
| KRT73 | 1 | 1 | 0.98 |
| FRZB | 1 | 1 | 0.97 |
| KIAA1210 | 1 | 1 | 0.96 |
| HLA-C | 1 | 1 | 0.96 |
| LRRC31 | 1 | 1 | 0.96 |
| CD34 | 1 | 1 | 0.96 |
| EGFL8 | 1 | 1 | 0.96 |
| CHD1L | 1 | 1 | 0.95 |
| AE000662.92 | 1 | 1 | 0.95 |
| KRT2 | 1 | 1 | 0.95 |
| C10orf2 | 1 | 1 | 0.94 |
| TGFBR2 | 1 | 1 | 0.94 |
| LONP2 | 1 | 1 | 0.94 |
| PGF | 1 | 1 | 0.94 |
| MATN3 | 1 | 1 | 0.94 |
| TFF3 | 1 | 1 | 0.94 |
| FAM3C | 1 | 1 | 0.94 |
| C9 | 1 | 1 | 0.93 |
| C8orf12 | 1 | 1 | 0.93 |
| MTRNR2L6 | 1 | 1 | 0.93 |
| AL049747.1 | 1 | 1 | 0.93 |
| DNMT3A | 1 | 1 | 0.93 |
| PISD | 1 | 1 | 0.92 |
| ANG | 1 | 1 | 0.92 |
| THOC3 | 1 | 1 | 0.92 |
| TUBA3D | 1 | 1 | 0.92 |
| C10orf91 | 1 | 1 | 0.92 |
| FAM73B | 1 | 1 | 0.91 |
| DMKN | 1 | 1 | 0.91 |
| UGT2B15 | 1 | 1 | 0.91 |
| RELL1 | 1 | 1 | 0.91 |
| AP1S1 | 1 | 1 | 0.91 |
| DSC1 | 1 | 1 | 0.91 |
| MTFR2 | 1 | 1 | 0.91 |
| KLHL2 | 1 | 1 | 0.9 |
| NUDT6 | 1 | 1 | 0.9 |
| CNOT4 | 1 | 1 | 0.9 |
| UHRF1BP1L | 1 | 1 | 0.9 |
| NAPG | 1 | 1 | 0.89 |
| AC074091.13 | 1 | 1 | 0.89 |
| ITGB1 | 1 | 1 | 0.89 |
| CST6 | 1 | 1 | 0.89 |
| SEMA3F | 1 | 1 | 0.88 |
| ZNF281 | 1 | 1 | 0.88 |
| LSM11 | 1 | 1 | 0.88 |
| RP11-242G20.1 | 1 | 1 | 0.88 |
| LINC01101 | 1 | 1 | 0.88 |
| NANOS3 | 1 | 1 | 0.88 |
| SYK | 1 | 1 | 0.88 |
| SLC26A9 | 1 | 1 | 0.88 |
| CLUL1 | 1 | 1 | 0.88 |
| AL136531.1 | 1 | 1 | 0.88 |
| FBXL5 | 1 | 1 | 0.87 |
| VAPB | 1 | 1 | 0.87 |
| LSM4 | 1 | 1 | 0.87 |
| SLC19A1 | 1 | 1 | 0.87 |
| ERP29 | 1 | 1 | 0.87 |
| PDCD4 | 1 | 1 | 0.85 |
| PDXK | 1 | 1 | 0.85 |
| NTSR2 | 1 | 1 | 0.85 |
| NPNT | 1 | 1 | 0.84 |
| MAPK6 | 1 | 1 | 0.84 |
| NUB1 | 1 | 1 | 0.84 |
| FAM168B | 1 | 1 | 0.84 |
| ADAD1 | 1 | 1 | 0.84 |
| GRB10 | 1 | 1 | 0.83 |
| IQGAP2 | 1 | 1 | 0.83 |
| COLEC10 | 1 | 1 | 0.83 |
| C8orf31 | 1 | 1 | 0.83 |
| ZNFX1 | 1 | 1 | 0.83 |
| PSAP | 1 | 1 | 0.83 |
| STAT4 | 1 | 1 | 0.82 |
| UBAC2 | 1 | 1 | 0.82 |
| ACBD6 | 1 | 1 | 0.82 |
| FGFBP2 | 1 | 1 | 0.82 |
| CEP95 | 1 | 1 | 0.82 |
| KLF5 | 1 | 1 | 0.82 |
| TRMT44 | 1 | 1 | 0.82 |
| C5orf27 | 1 | 1 | 0.82 |
| SALL4 | 1 | 1 | 0.81 |
| RP11-1085N6.3 | 1 | 1 | 0.81 |
| LAMTOR3 | 1 | 1 | 0.81 |
| QKI | 1 | 1 | 0.81 |
| NDUFB9 | 1 | 1 | 0.81 |
| RAB24 | 1 | 1 | 0.81 |
| ACAD8 | 1 | 1 | 0.81 |
| FAM102B | 1 | 1 | 0.81 |
| FUT4 | 1 | 1 | 0.81 |
| CCDC147 | 1 | 1 | 0.8 |
| ZNF366 | 1 | 1 | 0.8 |
| PC | 1 | 1 | 0.8 |
| PPFIBP2 | 1 | 1 | 0.8 |
| MRFAP1 | 1 | 1 | 0.8 |
| AC092964.1 | 1 | 1 | 0.79 |
| RPL37A | 1 | 1 | 0.79 |
| SC5D | 1 | 1 | 0.79 |
| NCK2 | 1 | 1 | 0.79 |
| FSCB | 1 | 0 | 3.31 |
| FAM110C | 1 | 0 | 3.05 |
| CDH10 | 1 | 0 | 2.76 |
| AKIRIN2 | 1 | 0 | 2.76 |
| PPP1R3C | 1 | 0 | 2.72 |
| LAMP5 | 1 | 0 | 2.57 |
| FAM107B | 1 | 0 | 2.52 |
| NAT2 | 1 | 0 | 2.45 |
| HTR2A | 1 | 0 | 2.27 |
| ACTRT1 | 1 | 0 | 2.23 |
| ACTL7A | 1 | 0 | 2.23 |
| ATP9B | 1 | 0 | 2.18 |
| TRPM7 | 1 | 0 | 2.16 |
| C20orf85 | 1 | 0 | 2.1 |
| LRRFIP2 | 1 | 0 | 2.08 |
| CYYR1 | 1 | 0 | 2.04 |
| IMPAD1 | 1 | 0 | 1.96 |
| SLC30A1 | 1 | 0 | 1.92 |
| INHBA | 1 | 0 | 1.9 |
| ZNF716 | 1 | 0 | 1.89 |
| SLITRK1 | 1 | 0 | 1.86 |
| GSTA3 | 1 | 0 | 1.85 |
| TET2 | 1 | 0 | 1.82 |
| POTED | 1 | 0 | 1.82 |
| SKIL | 1 | 0 | 1.81 |
| FRG1B | 1 | 0 | 1.77 |
| TRAM1L1 | 1 | 0 | 1.75 |
| UFL1 | 1 | 0 | 1.73 |
| CLDN23 | 1 | 0 | 1.68 |
| ADRB1 | 1 | 0 | 1.67 |
| YTHDC1 | 1 | 0 | 1.66 |
| LCA5 | 1 | 0 | 1.65 |
| MSL3 | 1 | 0 | 1.65 |
| GATA2 | 1 | 0 | 1.64 |
| SLC25A51 | 1 | 0 | 1.63 |
| FAM72D | 1 | 0 | 1.61 |
| LDOC1 | 1 | 0 | 1.61 |
| COMMD8 | 1 | 0 | 1.59 |
| POLR2K | 1 | 0 | 1.59 |
| INSL6 | 1 | 0 | 1.56 |
| TAL2 | 1 | 0 | 1.55 |
| CTR9 | 1 | 0 | 1.54 |
| SMEK1 | 1 | 0 | 1.51 |
| C12orf5 | 1 | 0 | 1.51 |
| CHAMP1 | 1 | 0 | 1.5 |
| ABRA | 1 | 0 | 1.48 |
| ANKRD33B | 1 | 0 | 1.47 |
| IL13RA2 | 1 | 0 | 1.47 |
| PLOD2 | 1 | 0 | 1.47 |
| TMEM212 | 1 | 0 | 1.46 |
| AC090574.1 | 1 | 0 | 1.46 |
| ZBED2 | 1 | 0 | 1.46 |
| DKFZP761K2322 | 1 | 0 | 1.45 |
| BZW1 | 1 | 0 | 1.45 |
| OXGR1 | 1 | 0 | 1.45 |
| MFAP3L | 1 | 0 | 1.44 |
| FBXW7 | 1 | 0 | 1.43 |
| PPA2 | 1 | 0 | 1.42 |
| TBX3 | 1 | 0 | 1.39 |
| ANXA1 | 1 | 0 | 1.39 |
| LPIN2 | 1 | 0 | 1.37 |
| IRX5 | 1 | 0 | 1.37 |
| RBM24 | 1 | 0 | 1.36 |
| DUSP5 | 1 | 0 | 1.34 |
| ANKRD50 | 1 | 0 | 1.34 |
| PAX1 | 1 | 0 | 1.34 |
| FAM71C | 1 | 0 | 1.34 |
| ZNF692 | 1 | 0 | 1.33 |
| MTERF | 1 | 0 | 1.33 |
| TAF1A | 1 | 0 | 1.33 |
| FABP5 | 1 | 0 | 1.32 |
| RP11-683L23.1 | 1 | 0 | 1.31 |
| AC099552.4 | 1 | 0 | 1.3 |
| AGPAT2 | 1 | 0 | 1.3 |
| METTL21A | 1 | 0 | 1.29 |
| AL590560.1 | 1 | 0 | 1.29 |
| VIM | 1 | 0 | 1.27 |
| DSCC1 | 1 | 0 | 1.26 |
| MC5R | 1 | 0 | 1.25 |
| MYEOV | 1 | 0 | 1.25 |
| RP11-796G6.2 | 1 | 0 | 1.24 |
| RP11-706O15.1 | 1 | 0 | 1.23 |
| PITX2 | 1 | 0 | 1.23 |
| TAX1BP1 | 1 | 0 | 1.22 |
| COA1 | 1 | 0 | 1.22 |
| MAPRE2 | 1 | 0 | 1.21 |
| ODC1 | 1 | 0 | 1.21 |
| JADE1 | 1 | 0 | 1.21 |
| BICD1 | 1 | 0 | 1.2 |
| LIF | 1 | 0 | 1.2 |
| ADAMTS18 | 1 | 0 | 1.2 |
| TRA2B | 1 | 0 | 1.2 |
| CD46 | 1 | 0 | 1.2 |
| INMT | 1 | 0 | 1.2 |
| AREG | 1 | 0 | 1.2 |
| ZBED5 | 1 | 0 | 1.19 |
| ZNF630 | 1 | 0 | 1.19 |
| LINC00346 | 1 | 0 | 1.18 |
| BNC1 | 1 | 0 | 1.18 |
| PCDH18 | 1 | 0 | 1.17 |
| CYR61 | 1 | 0 | 1.17 |
| C10orf113 | 1 | 0 | 1.17 |
| CDC42EP3 | 1 | 0 | 1.16 |
| PDP1 | 1 | 0 | 1.16 |
| HDDC2 | 1 | 0 | 1.15 |
| CDKN1B | 1 | 0 | 1.15 |
| FOXA2 | 1 | 0 | 1.14 |
| AL079342.1 | 1 | 0 | 1.13 |
| GPR18 | 1 | 0 | 1.13 |
| MURC | 1 | 0 | 1.13 |
| ATP6V1G1 | 1 | 0 | 1.12 |
| ISL2 | 1 | 0 | 1.12 |
| FGFBP3 | 1 | 0 | 1.12 |
| SRI | 1 | 0 | 1.11 |
| ASCL1 | 1 | 0 | 1.11 |
| CAP1 | 1 | 0 | 1.1 |
| CLPTM1L | 1 | 0 | 1.1 |
| RP11-171N4.2 | 1 | 0 | 1.1 |
| CRY2 | 1 | 0 | 1.1 |
| UBE2QL1 | 1 | 0 | 1.1 |
| PANK4 | 1 | 0 | 1.09 |
| PLSCR2 | 1 | 0 | 1.09 |
| BATF3 | 1 | 0 | 1.09 |
| SLITRK3 | 1 | 0 | 1.09 |
| FLG2 | 1 | 0 | 1.07 |
| DCAF4L2 | 1 | 0 | 1.07 |
| GPR6 | 1 | 0 | 1.07 |
| LRRN1 | 1 | 0 | 1.07 |
| ISL1 | 1 | 0 | 1.06 |
| RIPK2 | 1 | 0 | 1.06 |
| MYL12A | 1 | 0 | 1.06 |
| UBQLN1 | 1 | 0 | 1.06 |
| PARP1 | 1 | 0 | 1.06 |
| UTP23 | 1 | 0 | 1.05 |
| CASK | 1 | 0 | 1.05 |
| AP001421.1 | 1 | 0 | 1.05 |
| ARL14 | 1 | 0 | 1.05 |
| CCNT2 | 1 | 0 | 1.04 |
| CDKN2AIP | 1 | 0 | 1.04 |
| SLC35A3 | 1 | 0 | 1.04 |
| SYT10 | 1 | 0 | 1.03 |
| USP12 | 1 | 0 | 1.03 |
| FAHD2A | 1 | 0 | 1.03 |
| AC132192.1 | 1 | 0 | 1.03 |
| GPD2 | 1 | 0 | 1.02 |
| THBS1 | 1 | 0 | 1.02 |
| LGALS3 | 1 | 0 | 1.02 |
| NEUROD1 | 1 | 0 | 1.02 |
| UBP1 | 1 | 0 | 1.02 |
| PAF1 | 1 | 0 | 1.02 |
| C11orf96 | 1 | 0 | 1.01 |
| MMD | 1 | 0 | 1.01 |
| TLR2 | 1 | 0 | 1 |
| NAA38 | 1 | 0 | 1 |
| SUCLG1 | 1 | 0 | 0.99 |
| ITGB8 | 1 | 0 | 0.99 |
| ABHD13 | 1 | 0 | 0.99 |
| AIFM1 | 1 | 0 | 0.99 |
| SNRPE | 1 | 0 | 0.99 |
| FASLG | 1 | 0 | 0.99 |
| TMA16 | 1 | 0 | 0.99 |
| NAMPT | 1 | 0 | 0.98 |
| ETAA1 | 1 | 0 | 0.98 |
| TMEM229A | 1 | 0 | 0.98 |
| NOV | 1 | 0 | 0.97 |
| GDNF | 1 | 0 | 0.97 |
| FAM9C | 1 | 0 | 0.97 |
| TRAF1 | 1 | 0 | 0.97 |
| MAFB | 1 | 0 | 0.97 |
| RP11-116D17.1 | 1 | 0 | 0.97 |
| MC3R | 1 | 0 | 0.97 |
| RABL2A | 1 | 0 | 0.97 |
| C8orf33 | 1 | 0 | 0.97 |
| SAMD5 | 1 | 0 | 0.97 |
| C5orf47 | 1 | 0 | 0.95 |
| EIF4A2 | 1 | 0 | 0.95 |
| CNGB3 | 1 | 0 | 0.95 |
| ANKRD9 | 1 | 0 | 0.95 |
| OTOR | 1 | 0 | 0.95 |
| MTRNR2L13 | 1 | 0 | 0.95 |
| MKI67 | 1 | 0 | 0.94 |
| THOC2 | 1 | 0 | 0.94 |
| VLDLR | 1 | 0 | 0.94 |
| SDPR | 1 | 0 | 0.94 |
| CEBPA | 1 | 0 | 0.94 |
| MDH2 | 1 | 0 | 0.94 |
| KLHL1 | 1 | 0 | 0.94 |
| JMY | 1 | 0 | 0.94 |
| SFTPB | 1 | 0 | 0.94 |
| INSIG2 | 1 | 0 | 0.94 |
| RP11-89N17.1 | 1 | 0 | 0.94 |
| SLC12A2 | 1 | 0 | 0.94 |
| BUB3 | 1 | 0 | 0.93 |
| ARHGAP5 | 1 | 0 | 0.93 |
| FRG1 | 1 | 0 | 0.93 |
| RP11-204N11.1 | 1 | 0 | 0.93 |
| CTSD | 1 | 0 | 0.93 |
| PZP | 1 | 0 | 0.92 |
| AMER2 | 1 | 0 | 0.92 |
| FOXE1 | 1 | 0 | 0.92 |
| RP11-6L6.2 | 1 | 0 | 0.92 |
| KIAA1586 | 1 | 0 | 0.92 |
| ORC5 | 1 | 0 | 0.92 |
| AKR1D1 | 1 | 0 | 0.91 |
| NYX | 1 | 0 | 0.91 |
| HIST1H2BF | 1 | 0 | 0.91 |
| COX10 | 1 | 0 | 0.91 |
| NEURL1B | 1 | 0 | 0.91 |
| MRPS30 | 1 | 0 | 0.9 |
| KCNS2 | 1 | 0 | 0.9 |
| FAM122A | 1 | 0 | 0.9 |
| MARCO | 1 | 0 | 0.89 |
| SMIM15 | 1 | 0 | 0.89 |
| ZXDB | 1 | 0 | 0.89 |
| WBSCR27 | 1 | 0 | 0.88 |
| DAB2 | 1 | 0 | 0.88 |
| GADD45B | 1 | 0 | 0.88 |
| NT5E | 1 | 0 | 0.88 |
| C1orf52 | 1 | 0 | 0.88 |
| PRRC1 | 1 | 0 | 0.88 |
| RASA2 | 1 | 0 | 0.88 |
| EPCAM | 1 | 0 | 0.88 |
| NR2F1 | 1 | 0 | 0.88 |
| RP11-1407O15.2 | 1 | 0 | 0.88 |
| C2orf57 | 1 | 0 | 0.87 |
| WAS | 1 | 0 | 0.87 |
| TCEAL1 | 1 | 0 | 0.87 |
| TSSK1B | 1 | 0 | 0.87 |
| LAMP2 | 1 | 0 | 0.86 |
| CTD-2210P24.4 | 1 | 0 | 0.86 |
| REEP2 | 1 | 0 | 0.86 |
| PCBP1 | 1 | 0 | 0.86 |
| PTGS2 | 1 | 0 | 0.86 |
| CLN5 | 1 | 0 | 0.86 |
| NFKBIZ | 1 | 0 | 0.86 |
| TPRA1 | 1 | 0 | 0.86 |
| CYP26C1 | 1 | 0 | 0.86 |
| C13orf45 | 1 | 0 | 0.86 |
| KIAA0947 | 1 | 0 | 0.86 |
| NABP1 | 1 | 0 | 0.86 |
| DSEL | 1 | 0 | 0.85 |
| WASL | 1 | 0 | 0.85 |
| IRX1 | 1 | 0 | 0.85 |
| AFTPH | 1 | 0 | 0.85 |
| ING2 | 1 | 0 | 0.85 |
| INSM1 | 1 | 0 | 0.85 |
| HGSNAT | 1 | 0 | 0.85 |
| AC187652.1 | 1 | 0 | 0.85 |
| SLC38A4 | 1 | 0 | 0.85 |
| AC008948.1 | 1 | 0 | 0.85 |
| NKX2-2 | 1 | 0 | 0.85 |
| TOP2B | 1 | 0 | 0.85 |
| PSKH2 | 1 | 0 | 0.84 |
| BMP3 | 1 | 0 | 0.84 |
| AC107021.1 | 1 | 0 | 0.84 |
| ICOS | 1 | 0 | 0.84 |
| PPP1CB | 1 | 0 | 0.84 |
| SERP2 | 1 | 0 | 0.84 |
| AC022498.1 | 1 | 0 | 0.84 |
| ACP6 | 1 | 0 | 0.84 |
| AC007461.1 | 1 | 0 | 0.84 |
| CISH | 1 | 0 | 0.83 |
| LRRC37A3 | 1 | 0 | 0.83 |
| TACR3 | 1 | 0 | 0.83 |
| NUDT4 | 1 | 0 | 0.83 |
| MAGED2 | 1 | 0 | 0.83 |
| RNF149 | 1 | 0 | 0.83 |
| ASZ1 | 1 | 0 | 0.83 |
| SERHL2 | 1 | 0 | 0.83 |
| MIS18BP1 | 1 | 0 | 0.83 |
| PITRM1 | 1 | 0 | 0.83 |
| FZD10 | 1 | 0 | 0.83 |
| SOCS2 | 1 | 0 | 0.83 |
| PIEZO2 | 1 | 0 | 0.82 |
| CBWD2 | 1 | 0 | 0.82 |
| LRP5L | 1 | 0 | 0.81 |
| MRPL42 | 1 | 0 | 0.81 |
| SRP14 | 1 | 0 | 0.81 |
| OLFML3 | 1 | 0 | 0.81 |
| MSL2 | 1 | 0 | 0.81 |
| RPS3A | 1 | 0 | 0.81 |
| ASCL4 | 1 | 0 | 0.81 |
| SLC28A3 | 1 | 0 | 0.81 |
| MARC1 | 1 | 0 | 0.81 |
| IL1F10 | 1 | 0 | 0.81 |
| NPY5R | 1 | 0 | 0.81 |
| TMEM17 | 1 | 0 | 0.81 |
| MGST1 | 1 | 0 | 0.81 |
| PFKL | 1 | 0 | 0.81 |
| AGFG1 | 1 | 0 | 0.81 |
| VTCN1 | 1 | 0 | 0.8 |
| MGAT4A | 1 | 0 | 0.8 |
| RP11-160N1.10 | 1 | 0 | 0.8 |
| ARHGAP29 | 1 | 0 | 0.8 |
| ANKRD60 | 1 | 0 | 0.8 |
| TRIM48 | 1 | 0 | 0.8 |
| EOMES | 1 | 0 | 0.8 |
| AKTIP | 1 | 0 | 0.8 |
| SOD3 | 1 | 0 | 0.8 |
| N6AMT1 | 1 | 0 | 0.8 |
| AGTR2 | 1 | 0 | 0.79 |
| CCDC122 | 1 | 0 | 0.79 |
| EGR3 | 1 | 0 | 0.79 |
| RFK | 1 | 0 | 0.79 |
